# IL-5 CAR-T cell therapy induces effective remission in hypereosinophilic disorders

**DOI:** 10.1186/s13045-026-01782-x

**Published:** 2026-02-13

**Authors:** Youqian Wu, Ruiqi Zhang, Beibei Sun, Gaoying Chen, Fang Xu, Xinyi Chen, Jiayuan Zeng, Dan Shen, Fan Shi, Sheng Pan, Bingpeng Yao, Haoyu Tang, Zhehua Shao, Qian Wu, Jiawei Shao, Chao Zhang, Dongrui Wang, Yongxian Hu, Songmin Ying

**Affiliations:** 1 Department of Pharmacy, Center for Regeneration and Aging Medicine, the Fourth Affiliated Hospital of School of Medicine, and International School of Medicine, International Institutes of Medicine, Zhejiang University, Zhejiang-Denmark Joint Laboratory of Regeneration and Aging Medicine, Yiwu, 322000 China; 2 Key Laboratory of Respiratory Disease of Zhejiang Province, Department of Respiratory and Critical Care Medicine, The Second Affiliated Hospital of Zhejiang University School of Medicine, Hangzhou, 310009 China; 3 Department of Pharmacology, Zhejiang University School of Medicine, Hangzhou, 310058 China; 4 Bone Marrow Transplantation Center of The First Affiliated Hospital & Liangzhu Laboratory, Zhejiang University School of Medicine, Hangzhou, 310003 China

**Keywords:** Hypereosinophilic syndrome, Chronic eosinophilic leukemia, Eosinophils, CAR-T cell therapy, IL-5Rα, Preclinical efficacy, Preclinical safety

## Abstract

**Background:**

Clonal and pathogenic eosinophil expansion in hypereosinophilic disorders (e.g., refractory hypereosinophilic syndrome (HES) and chronic eosinophilic leukemia (CEL)) remains an unmet therapeutic challenge, with current strategies often failing to induce durable remission. While monoclonal antibodies targeting the IL-5/IL-5Rα pathway have shown efficacy in treating eosinophil-driven diseases, a subset of patients experience incomplete responses or relapses, highlighting the need for more durable and comprehensive therapeutic strategies. Chimeric antigen receptor T (CAR-T) cell therapy, with its potential for long-term persistence and durable remission after a single infusion, represents a promising alternative for patients with refractory disease.

**Methods:**

We performed single-cell RNA sequencing on peripheral blood (PB) and bone marrow (BM) samples from both healthy individuals and the patient with hypereosinophilic disorder, to identify key therapeutic targets for intervention. Based on these findings, we developed a first-in-class CAR-T therapy using human interleukin-5 (hIL-5) as a ligand-based targeting domain, to selectively recognize and eliminate IL-5Rα^+^ eosinophils and precursors. In vitro cytotoxicity and IFN-γ secretion were measured against target cells and patient-derived PB/BM samples. Preclinical safety was evaluated through comprehensive toxicity assessment. Efficacy was evaluated in a hypereosinophilic leukemia mouse model, with tumor burden reduction and survival as the key evaluation indicators.

**Results:**

Single-cell profiling revealed concurrent expansion of both eosinophil progenitors and mature eosinophils in the BM and PB, highlighting the need for therapies targeting all stages of eosinophil development. IL-5 receptor α (IL-5Rα) was identified as the optimal target due to its high expression across all stages of eosinophil development, with relatively restricted expression on non-eosinophil immune populations. hIL-5 CAR-T cells demonstrated potent in vitro cytotoxicity and IFN-γ secretion against target cells and effectively eliminated eosinophils in patient-derived PB/BM samples. No dose-limiting toxicities were observed, and no evidence of cytokine release syndrome (CRS) was detected in preclinical models. In the hypereosinophilic leukemia mouse model, a single infusion of hIL-5 CAR-T cells significantly reduced tumor burden and extended survival, demonstrating its therapeutic potential.

**Conclusions:**

IL-5 CAR-T cell therapy represents a promising targeted therapeutic approach for IL-5Rα-expressing hypereosinophilic disorders. Its ability to target eosinophils and their precursors across all developmental stages in BM and PB addresses a critical unmet medical need in refractory HES and CEL.

**Supplementary Information:**

The online version contains supplementary material available at 10.1186/s13045-026-01782-x.

## Introduction

Hypereosinophilic syndrome (HES) is a rare hematologic disorder characterized by sustained peripheral blood eosinophilia [1], leading to organ damage mediated by eosinophil infiltration and cytotoxic mediator release [2]. Chronic eosinophilic leukemia (CEL), a clonal myeloid neoplasm related to HES, is characterized by autonomous eosinophil proliferation and remains a therapeutic challenge [3]. Current treatments often provide only transient symptom relief; however, their limited efficacy and substantial adverse effects underscore the need for more effective therapeutic strategies [4–6]. Dysregulated interleukin-5 (IL-5) signaling promotes eosinophil survival and proliferation by interacting with the interleukin-5 receptor alpha (IL-5Rα, CD125). This process drives disease progression in hypereosinophilic disorders mediated by cytokines [7, 8]. Thus, targeted inhibition of the IL-5/IL-5Rα pathway emerges as a promising therapeutic strategy to address the limitations of conventional therapies and improve clinical outcomes for selected patients with treatment-refractory hypereosinophilic disorders [9, 10].

Several monoclonal antibodies targeting the IL-5/IL-5Rα pathway have demonstrated promising clinical efficacy in trials for treating selected eosinophil-driven diseases [11–14]. However, a subset of patients experience incomplete responses, relapse after discontinuation, or persistent disease driven by underlying clonal eosinophil progenitors, highlighting the need for more durable and comprehensive therapeutic strategies [15, 16].

Chimeric antigen receptor T (CAR-T) cell therapy has revolutionized the treatment of hematological malignancies. B-cell-targeted approaches, particularly those directed against CD19, are the most extensively studied and clinically validated [17]. FDA-approved CD19-targeted CAR-T products, such as tisagenlecleucel and axicabtagene ciloleucel, have demonstrated impressive efficacy in inducing remission and prolonging survival in patients with B-cell acute lymphoblastic leukemia (B-ALL) and B-cell non-Hodgkin lymphoma (B-NHL) [18, 19]. This success highlights the potential of CAR-T technology to target other cell types involved in hematological disorders. Monoclonal antibodies require repeated administration to maintain therapeutic efficacy, whereas CAR-T cells possess the capacity for in vivo expansion and can theoretically provide long-term activity. This property provides a rationale for investigating their potential in achieving more sustained disease control following a single infusion. Consequently, CAR-T therapy represents a novel investigational approach, potentially applicable for patients with refractory HES or CEL who do not achieve adequate control with conventional treatments.

In this study, single-cell profiling of an HES patient revealed synchronous expansion of eosinophil progenitors and mature eosinophils within BM, a previously underappreciated feature that underscores the need for pan-developmental stage targeting across both BM and PB. IL-5Rα was selected as an optimal therapeutic target based on its stage-unrestricted expression across eosinophil developmental stages and its relatively restricted expression among other immune cell populations. Using human IL-5 as a ligand-based antigen recognition domain, we developed a novel CAR-T strategy designed to target IL-5Rα-expressing eosinophils and their progenitors, with the potential to ameliorate disease manifestations in HES and CEL. Rigorous preclinical evaluations demonstrated that human IL-5 CAR-T cells exhibit significant efficacy and a favorable safety profile in mouse models of hypereosinophilic leukemia. These findings provide a rationale for the clinical translation of hIL-5 CAR-T cell therapy in severe hypereosinophilic disorders and suggest its potential applicability in selected, refractory eosinophil-associated diseases, such as asthma and allergic bronchopulmonary aspergillosis (ABPA), which share the pathological feature of eosinophil-mediated tissue damage.

## Methods

### Study design

This study performed a comprehensive single-cell analysis of bone marrow (BM) and peripheral blood (PB) from the HES patient versus healthy donors, revealing key disease pathogenic mechanisms and identifying pathological target cells. Subsequent comprehensive receptor profiling identified the optimal therapeutic target for ablating pathogenic eosinophils across developmental stages. The goal of this study was to evaluate the therapeutic potential of hIL-5 CAR-T cell therapy for the treatment of hypereosinophilic disorders. To bridge preclinical discovery with therapeutic applications, we established a Good Manufacturing Practice (GMP)-grade manufacturing process for clinical-grade hIL-5 CAR-T cells under optimized conditions to ensure product consistency and regulatory compliance. In vitro functional assessments included cytotoxicity evaluation through luminescent cell viability assays and IFN-γ secretion profiling. For in vivo safety evaluation, two models were employed: (1) Tumorigenicity was assessed in 6–8-week-old BALB/c nude mice (*n* = 5–6/group) or NCG mice (*n* = 10/group) through tumor volume monitoring; (2) Systemic toxicity was investigated in 4–5-week-old NCG mice (5 females and 5 males/group) through histopathological analysis of major organs, organ-to-body weight ratio quantification, inflammation scoring, and serum cytokine profiling via multiplex immunoassays. Anti-tumor efficacy was evaluated in 6–8-week-old NCG mice bearing hIL-5Rα-expressing Nalm6 xenografts (*n* = 6/group) or K562 xenografts (*n* = 5–6/group). Tumor progression was tracked using bioluminescent imaging, with parallel monitoring of survival outcomes and weight changes. Detailed information regarding the number of replicates, the statistical test used, and the corresponding P values is provided in the figure legends.

### Antibodies

To assess hIL-5 CAR expression on hIL-5 CAR-T cells, we used APC anti-mouse/human IL-5 antibody (BioLegend) and FITC anti-human CD3 antibody (BioLegend). To detect hIL-5Rα expression in hIL-5Rα-overexpressing cell lines, we used PE anti-human CD125 (IL-5Rα) antibody (BioLegend). To detect the activation of the hIL-5 CAR-T cells, we used PE anti-human CD69 antibody (BioLegend). For cell proportion analysis of eosinophils, neutrophils, and basophils, flow cytometry was performed using the following antibodies and reagents: PerCP/Cyanine5.5 anti-human CD45 antibody (BioLegend), APC anti-human Siglec-8 antibody (BioLegend), PE anti-human CD125 (IL-5Rα) antibody (BioLegend), PE/Dazzle 594 anti-human CD193 (CCR3) antibody (BioLegend), APC-A750 anti-human CD14 antibody (BioLegend), and BV605 anti-human CD15 antibody (BioLegend).

### Mice and cell lines

NCG mice (NOD/ShiLtJGpt-*Prkdc*^em26Cd52^*Il2rg*^em26Cd22^/Gpt, Strain NO. T001475) and nude mice (BALB/cNj-Foxn1^nu^/Gpt, Strain NO. D000521) were purchased from GemPharmatech Co., Ltd. All animal experiments were strictly conducted following the protocols approved by the Ethics Committee for Animal Studies at Zhejiang University, China (ZJU20240022). Age- and sex-matched mice were used in all experiments. In all animal experiments, n denotes the number of mice per experimental group, unless otherwise specified.

U-2 OS, HeLa, MRC-5, and HEK293T cells were cultured in Dulbecco’s modified Eagle’s medium (DMEM, BioChannel) supplemented with 10% fetal bovine serum (FBS, Corning) and 100 IU/mL penicillin and streptomycin (10 mg/mL, Gibco). K562 and Nalm6 cells were cultured in RPMI-1640 medium (BioChannel) supplemented with 10% FBS and 100 IU/mL penicillin and streptomycin.

### Single cell transcriptome capture, library construction and sequencing

Cells were first stained with two fluorescent dyes, Calcein AM and DRAQ7, to precisely determine cell concentration and viability using a BD Rhapsody Scanner (BD Biosciences). Cells were loaded into a BD Rhapsody microwell cartridge as previously described by Fan et al. [20]. Cell capture beads were then loaded in excess to ensure that nearly every microwell contained a single bead, and excess beads were washed away. Following cell lysis, cell capture beads were retrieved and washed prior to reverse transcription. Single-cell transcriptomes captured on microbeads were converted into cDNA libraries containing cell barcodes and unique molecular identifiers (UMIs). All procedures were performed using the BD Rhapsody cDNA Kit (BD Biosciences, Cat. No. 633773) and BD Rhapsody Targeted mRNA & AbSeq Amplification Kit (BD Biosciences, Cat. No. 633801) according to the manufacturer’s instructions. Libraries were sequenced in paired-end 150-bp mode (PE150) on an Illumina NovaSeq platform.

### scRNA-seq data pre-processing and quality control

For scRNA-seq data analysis, the BD Rhapsody Sequence Analysis Pipeline (version 2.3) was used to align sequencing reads and generate gene-cell unique molecular identifier (UMI) count matrices for each sample. Reads were mapped to the human reference genome (GRCh38). Bone marrow (BM) donor datasets were obtained from the Gene Expression Omnibus (GEO) under accession number GSE120221 [21]. Downstream analyses were performed using the Seurat R package (version 4.3) [22]. Lowly expressed genes, defined as genes detected in ≤ 10 cells, were filtered out. To remove low-quality cells and potential doublets, cells were excluded based on the following criteria: [1] fewer than 400 or more than 6,000 detected genes; [2] fewer than 500 or more than 50,000 UMIs; [3] mitochondrial UMIs accounting for more than 15% of total UMIs; and [4] a log10-transformed gene-to-UMI ratio (log10GenesPerUMI) of less than 0.8. Cell doublets were identified using Scrublet (v1.0) [23].

### scRNA-seq data integration, dimensionality reduction, clustering, and visualization

Seurat v4.3 was used for subsequent clustering analysis and visualization. Raw counts for the filtered cells were normalized by ‘NormalizeData’ function. For clustering the cells, top 2000 variable features were selected and their normalized expression was scaled. We removed the batch effect across different individuals by identifying anchors between individuals and passing these anchors to the ‘IntegrateData’ function of Seurat R packages. For visualization, the dimensionality was further reduced using UMAP. To cluster single cells based on their expression profiles, we used an unsupervised graph-based clustering algorithm, Louvain. Major cell type annotation was conducted by leveraging canonical marker genes (e.g., *IL5-RA*, *SMPD3*, *SIGLEC8* and *CCR3*). As to the eosinophil sub-clustering, the same process was performed.

### Cell proportion change calculation

For each sample, the proportion of a specific cell type was calculated by dividing the number of cells belonging to that cell type by the total number of cells in the sample. This process was performed for each major cell type across all samples to generate a proportion matrix. As to the sub cell population, the proportion of a specific sub-type was calculated by dividing the number of cells by the total number of this major cell in the sample. Then the changes in cell proportions were visualized using stacked bar plots.

### hIL-5 CAR plasmid construction and lentivirus production

The hIL-5 CAR was engineered using a lentiviral transfer vector, composed of the human CD8α signal peptide fused to human IL-5, human CD8α hinge and transmembrane domains, followed by the human CD28 co-stimulatory domain and the human CD3ζ signaling domain. For lentivirus production, the hIL-5 CAR lentiviral plasmid and the helper plasmids pMD2.G and psPAX2 were co-transfected into HEK293T cells using polyethylenimine (PEI, Yeasen). The supernatant was collected 48 h after transfection, filtered through 0.45-µm filters (Millipore), and stored at −80 °C.

### Methods for the preparation of hIL-5 CAR-T cells at GMP levels [24]

#### Day 0: T cell enrichment and activation

Preparation of Enrichment Buffer: To prepare a 50 mL working solution, combine 47.5 mL of PBS/EDTA buffer with 2.5 mL of 20% human serum albumin (HSA) to achieve a final concentration of 1% HSA.

Thawing of PBMCs: Retrieve cryopreserved PBMCs from the liquid nitrogen tank and place them in a 37 °C water bath. Thaw the PBMCs with gentle swirling until only a few ice crystals remain. Immediately transfer the thawed PBMCs to pre-warmed complete medium, X-VIVO 15 serum-free medium, supplemented with 2% immune cell serum replacement and 1000 IU/mL of IL-2. Centrifuge at 400×g for 10 min, discard the supernatant, and resuspend the cells in pre-warmed complete medium. Perform a viable cell count.

Calculation and Enrichment: Estimate the theoretical yield of CD3^+^ T cells based on the viable cell count and CD3^+^ percentage provided in the PBMC certificate of analysis (CoA). Pellet the PBMCs by centrifugation at 300×g for 10 min. Discard the supernatant, and resuspend the cells in the enrichment buffer at a density of 1 × 10^8^ cells/mL. Add CliniMACS CD3 Reagent at a ratio of 5 µL per 1 × 10^7^ estimated CD3^+^ T cells. Gently mix and incubate in a 6-well plate on a rocking platform (30 rpm) at room temperature for 30 min, protected from light.

Cell sorting was performed following the manufacturer’s instructions. Briefly, load the LS column onto the Miltenyi MACS magnetic separator. Rinse the LS column with 3 mL of enrichment buffer. Slowly add the cell suspension to the center of the LS column membrane, avoiding air bubbles. Allow the cells to pass through the column with CD3^+^ T cells retained. Wash the column three times with 3 mL of enrichment buffer. After washing, remove the LS column from the magnetic rack and elute the magnetically labeled CD3^+^ T cells by adding 5 mL of enrichment buffer.

Activation of T Cells: Resuspend the sorted T cells in complete medium to a final concentration of 2.86 × 10^6^ cells/mL. Add TransAct CD3/CD28 reagent at a 1:17.5 ratio (culture volume: Transact volume) and transfer the cells to a 6-well plate. Incubate at 37 °C with 5% CO_2_ for activation.

#### Day 1: lentivirus transduction

Retrieve the lentivirus and thaw it in the dark at 2–8 °C. Remove the cultured cells from the CO_2_ incubator, take a sample for counting, and centrifuge at 400×g for 10 min. Discard the supernatant and resuspend the cells in complete medium (X-VIVO 15 serum-free medium, 2% Immune Cell Serum Replacement, 1000 IU/mL IL-2) to a density of 4 × 10^6^ cells/mL. Calculate the required lentivirus volume using the formula: Lentivirus Volume (mL) = (Total Number of Viable Cells×MOI)/Lentiviral Titer (TU/mL). Add the lentivirus at an MOI of 2, and then supplement with complete medium to achieve a final cell density of 1.5 × 10^6^ cells/mL. Transfer the cells to a 6-well plate and equilibrate. Place the plate in a centrifuge at 20 °C, 1000×g, with an acceleration rate of 3 and deceleration rate of 3. Incubate at 37 °C with 5% CO_2_.

#### Day 2: lentivirus removal

24 h post-transduction, collect samples for cell counting. Centrifuge at 400×g for 10 min, discard the supernatant, and resuspend the cells in complete medium to a final concentration of 5 × 10^5^ cells/mL. Transfer the cells to a 6-well plate and incubate at 37 °C with 5% CO_2_.

#### Day 5, day 8, day 10, day 12: cell counting and testing

Take samples for counting, and perform CAR positivity testing by flow cytometry. Adjust the cell density to 5 × 10^5^ cells/mL using complete medium and transfer the cells to culture flasks for further incubation at 37 °C with 5% CO_2_. On day 10, if the cells meet the harvest criteria (CAR-T viable cell count reached ≥ 1.3 × 10^9^), cryopreserve them 1 × 10^7^ CAR-T cells/mL, with 1 mL per vial. If the cells do not meet the harvest criteria on Day 10, harvest them on Day 12, provided they meet the same viable cell count threshold.

### Cryopreservation

If cells are harvested, resuspend them in CryoStor CS10 to a final concentration of 1 × 10^7^ CAR-T cells/mL and aliquot into cryovials, with 1 mL per vial. Store the cryovials in a cryopreservation box at ≤−130 °C.

### Data analysis

Data and testing diagrams generated during the experiment were compiled into a comprehensive report.

### Cytotoxic activity assay

Cytotoxicity of MOCK-T cells and hIL-5 CAR-T cells was assessed using a luciferase-based assay as described previously [25, 26]. In detail, 2 × 10^4^ target cells stably expressing firefly luciferase were co-cultured with effector cells at the indicated effector-to-target (E:T) ratios in white 96-well plates (Costar). After indicated incubation time of co-culture, 100 µL of PBS containing 45 µg D-luciferin (GoldBio) was added to the medium. Luminescence was measured using a plate reader (SynergyMx M5, Molecular Devices). Target cells alone were plated at the same cell density to determine maximal luciferase expression (relative light units, RLU). Cytotoxicity was calculated according to the average loss of luminescence of the experimental condition relative to the control wells containing target cells.

For IFN-γ secretion assay, the culture supernatants were collected and centrifuged at 400×g for 10 min at room temperature. The supernatant was then transferred to new tubes for cytokine quantification using an IFN-γ-specific ELISA kit (R&D Systems), following the manufacturer’s instructions.

### Cytotoxic activity assay for CAR-T cells with patient-derived peripheral blood and bone marrow

PB was collected from the patient, and granulocytes, including neutrophils, eosinophils, and basophils, were isolated by Percoll density gradient centrifugation. After red blood cell lysis, granulocytes were counted, centrifuged, and resuspended in complete RPMI-1640 medium supplemented with 100 ng/mL IL-5 and 50 ng/mL G-CSF. The granulocytes were seeded into 48-well plates at a density of 1 × 10^5^ cells per well. CAR-T or MOCK-T cells were then added at the specified ratios, and the co-cultures were incubated at 37 °C with 5% CO_2_ for 24 h. After incubation, the co-cultured cells were collected, and flow cytometry was performed to evaluate the proportion of CD125-positive cells, eosinophils, and neutrophils.

BM cells were obtained via BM aspiration and used directly for co-culture without the need for Percoll density gradient separation. The co-culture procedure for BM samples was identical to that for PB samples.

This study was approved by the Clinical Research Ethics Committee of the First Affiliated Hospital, Zhejiang University School of Medicine, under the ethics approval number [2025B] IIT Ethics Approval NO.0743 and conducted in accordance with the Declaration of Helsinki. Informed consent was obtained from all participants, and their privacy and confidentiality were strictly maintained throughout the study.

### Generation of hIL-5Rα-overexpressing (OE) stable cell lines

The full-length human hIL-5Rα coding sequence was cloned into the pLVX-puro vector. For lentivirus packaging, the helper plasmid psPAX2, pMD2.G and the hIL-5Rα plasmid were co-transfected into HEK293T cells using PEI-mediated transfection. Viral supernatants were harvested at 48–72 h post-transfection and filtered through 0.45-µm filters. U-2 OS, Nalm6 and K562 cells were transduced with hIL-5Rα lentivirus. 48 h post-infection, expression efficiency was evaluated by flow cytometry.

### Mouse xenograft tumor model

Female NCG mice aged six to eight weeks were used. For the Nalm6 model, 1 × 10^6^ F-Luc^+^ IL-5Rα-Nalm6 cells were injected intravenously [27]. Seven days later, mice were randomly assigned (*n* = 6 per group), and MOCK-T or CAR-T cells were injected intravenously. Tumor burden was assessed using the Optical imaging system (MiLabs) after intraperitoneal injection of 100 µL 150 mg/mL D-luciferin. Body weight was monitored, and mice were observed for signs of health deterioration. Mice were euthanized when the physical or behavioural health declined below established thresholds. When the established thresholds or body weight dropped by more than 20%, mice were euthanized.

For the K562 model, 5 × 10^6^ F-Luc^+^ IL-5Rα-K562 cells were injected intravenously. CAR-T or MOCK-T cells were subsequently infused, and tumor burden, body weight, and health status were monitored following the same protocol as the Nalm6 model.

### IL-33 alarmin-induced eosinophilia model

Six-to-eight-week-old female BALB/c mice were injected intraperitoneally with 400 ng of recombinant mouse IL-33 (Novoprotein Recombinant Mouse IL-33, Cat. No. CG73) in sterile PBS daily for 7 days. For the treatment group, mice received a tail vein injection of either MOCK-T or mouse IL-5 (mIL-5) CAR-T cells, administered either 5 days before or on the day of the initial IL-33 challenge. On Day 12, mice were euthanized, and BM, PB, and spleen were collected for the analysis of eosinophil proportions and numbers.

### Soft agar colony formation assay

The soft agar colony formation assay was organized into six groups: a blank control group (no cells), a negative control group (1000 cells/well of MRC-5 cells), a positive control group (1000 cells/well of HeLa cells), a hIL-5 CAR-T low-dose group (1000 cells/well), a hIL-5 CAR-T medium-dose group (2000 cells/well), and a hIL-5 CAR-T high-dose group (4000 cells/well). Cells were seeded in 6-well plates with a bottom layer of 1.2% agar and an overlay of 0.7% agar containing the respective cell suspensions. After two weeks of culture, colony formation was observed, stained with crystal violet, and colonies were counted to assess tumorigenic potential [28].

### Tumorigenicity assay

Seven-to-eight-week-old male nude mice or NCG mice were divided into five groups: MRC-5 cells group (negative control), HeLa cells group (positive control), and low-, medium- and high-dose groups of hIL-5 CAR-T cells. The test cells were subcutaneously inoculated into the mice, and the inoculation site was monitored for progressive nodule development over a period of at least 16 weeks. At the end of the observation period, cells that failed to form progressive nodules were deemed non-tumorigenic. The termination criteria for euthanizing the mice included tumor volume exceeding 2000 mm^3^. All animals were euthanised, and the injection site, along with other organs (e.g., heart, lung, liver, spleen, kidney, brain) was examined histopathologically.

### In vivo biosafety assay

Four-to-five-week-old NCG mice were randomly divided into five groups (*n* = 10 per group, five females and five males) and received intravenous injections of either saline (Blank), T-cell cryopreservation solution (Vehicle), 1 × 10^6^ MOCK-T cells, a low dose of 1 × 10^6^ hIL-5 CAR-T cells, or a high dose of hIL-5 CAR-T cells (1 × 10^7^). Body weight was recorded weekly, and mice were euthanized on either day 8 or day 29 for subsequent evaluations based on the following parameters [29]:

#### Systemic toxicity evaluation

Body weight changes were monitored in all groups.

#### Organ toxicity evaluation

The heart, liver, spleen, lung, kidney, stomach, brain, and femur were harvested and examined for morphological changes. Each organ was weighed, and the organ-to-body weight ratio was calculated. Hematoxylin and eosin (H&E) staining was performed on major tissues (Hangzhou Boerfu Biotechnology Co., Ltd.) to assess inflammation and tissue damage, in accordance with the guidelines of the International Harmonization of Nomenclature and Diagnostic Criteria (INHAND).

#### Injection site evaluation

On day 29, the injection sites on the tails of all mice were examined for signs of infection, hematoma, or skin thickening.

#### Hematological analysis

PB samples were collected and analyzed for hematological parameters at the Research Center for Drug Safety Evaluation, Zhejiang University.

**Serum cytokines detection** 

Serum cytokine levels were quantified using the RayPlex^®^ Human Inflammation Array Kit (FAH-INF-1; RayBiotech, Peachtree Corners, GA, USA), a bead-based multiplex immunoassay capable of detecting 13 inflammatory proteins, including IFN-γ, IL-4, IL-6, IL-10, IL-17α, TNF-α, IL-1β, IL-2, IL-12p70, G-CSF, IL-13, IL-23p19 and MCP-1. The assay was conducted according to the manufacturer’s instructions. Briefly, serum samples were initially diluted 1:2 in PBS, followed by a 1:4 dilution upon mixing with antibody-conjugated capture beads. Lyophilized protein standards were reconstituted in serum diluent and serially diluted to establish a standard curve. The assay involved sequential incubation of the samples with a multiplex cocktail of antibody-conjugated beads, biotinylated detection antibody cocktail, and streptavidin-PE, with washing steps performed between each incubation. Bead fluorescence was analyzed by flow cytometry, and cytokine concentrations were calculated from mean fluorescence intensity (MFI) values using a five-parameter logistic regression model. Data analysis was conducted using FACSDiva^®^ Batch Analysis software, with final concentrations normalized to total dilution factors.

### Immunohistochemistry

After dissection, the tissues were fixed in 4% paraformaldehyde at 4 °C overnight. Following dehydration, the tissues were embedded in paraffin. Sections (5 μm) were mounted on slides, and antigen retrieval was performed using EDTA buffer (pH 9.0) with high temperature and pressure for 1 min 30 s. After cooling, the sections were incubated with primary antibodies: anti-human CD3 (ZSGB-Bio, ZA-0503), anti-human IL-5 (ArigoBio, ARG66000), followed by incubation with secondary antibody (goat anti-rabbit, Abcam). The sections were then visualized with an appropriate chromogen and imaged under a microscope.

### Cytometry by time-of-flight (CyTOF)

**Single cell suspension preparation from whole blood**: Blood sample: EDTA-treated whole blood was used to isolate peripheral blood mononuclear cells (PBMCs) via Ficoll density gradient centrifugation, following the standard operating procedure. After separation, 5 mL of cold Cell Staining Buffer (CSB) was added to resuspend the cell pellet. The cells were then collected by centrifugation at 300×g for 5 min at 2–8 °C. Following centrifugation, the supernatant was aspirated, and the cell pellet was resuspended in CSB. The cell concentration was determined by counting the cells. The prepared cells were then ready for further staining procedures. Tissue sample: The tissue was removed from the tissue storage solution and washed twice with cell culture medium. The tissue was then cut into 1 mm^3^ pieces and transferred into a C tube. A digestive enzyme mix (Miltenyi Biotec Human/Mouse Tumor Dissociation Kit or CHD) was added, and the volume was adjusted with cell culture medium to a final volume of 5 mL. The tissue mixture was incubated in a shaking incubator at 37 °C for 1 h to dissociate the tissue. After incubation, the dissociated tissue was filtered through a 70 μm cell strainer to remove debris. The cells were collected by centrifugation at 300×g for 5 min at 2–8 °C. The supernatant was aspirated, and the cell pellet was resuspended in CSB before counting the cell number. The prepared cells were then ready for further staining.

#### Mass cytometry staining, data acquisition

Cells were washed once with 1×PBS and then stained with 100 µL of 250 nM cisplatin (Fluidigm) for 5 min on ice to exclude dead cells, and then incubated in Fc receptor blocking solution before being stained with surface antibodies cocktail for 30 min on ice. Cells were washed twice with FACS buffer (1×PBS + 0.5% BSA) and fixed in 200 µL of intercalation solution (Maxpar Fix and Perm Buffer containing 250 nM ^191^/^193^Ir, Fluidigm) overnight. After fixation, cells were washed once with FACS buffer and then perm buffer (eBioscience), stained with intracellular antibodies cocktail for 30 min on ice. Cells were washed and resuspended with deionized water, adding into 20% EQ beads (Fluidigm), acquired on a mass cytometer (Helios, Fluidigm).

#### CyTOF data analysis

The data from each sample were debarcoded from the raw data using a doublet-filtering scheme with unique mass-tagged barcodes. Each .fcs file generated from different batches was normalized using the bead normalization method to ensure consistency across samples. Data were then manually gated using FlowJo software to exclude debris, dead cells, and doublets, retaining only live, single immune cells. The X-shift clustering algorithm was applied to partition the cells into distinct phenotypes based on their marker expression profiles. Each cluster was annotated according to its specific marker expression pattern, which was visualized on a heatmap of clusters versus markers. Dimensionality reduction was performed using the t-SNE algorithm to project the high-dimensional data into two dimensions. This visualization enabled the distribution of each cluster and the corresponding marker expression, highlighting differences between groups or sample types. Finally, statistical analysis was conducted using a t-test to assess the frequency of annotated cell populations across different conditions.

### Statistical analysis

All quantitative data are presented as the mean ± standard deviation (SD) or mean ± standard error of the mean (SEM) from at least three independent experiments. The specific number of biological replicates (n) for each experiment is provided in the corresponding figure legends. Statistical analyses were performed using GraphPad Prism software (8.0). Based on the experimental design: Comparisons between two groups were analyzed using an unpaired, two-tailed Student’s t-test. Comparisons among multiple groups with one independent variable were analyzed by one-way analysis of variance (ANOVA), followed by an appropriate post-hoc test (e.g., Tukey’s or Sidak’s) for multiple comparisons. Comparisons among multiple groups with two independent variables were analyzed by two-way ANOVA, followed by an appropriate post-hoc test for multiple comparisons. The specific statistical test applied to each dataset is explicitly stated in the respective figure legends. P value of less than 0.05 was considered statistically significant, and significance levels are denoted as follows: *P < 0.05, **P < 0.01, ***P < 0.001, ****P < 0.0001; ns (not significant) indicates *P* > 0.05.

## Results

### Single-cell profiling characterizes human eosinophils in hypereosinophilic syndrome

**Fig. 1:**
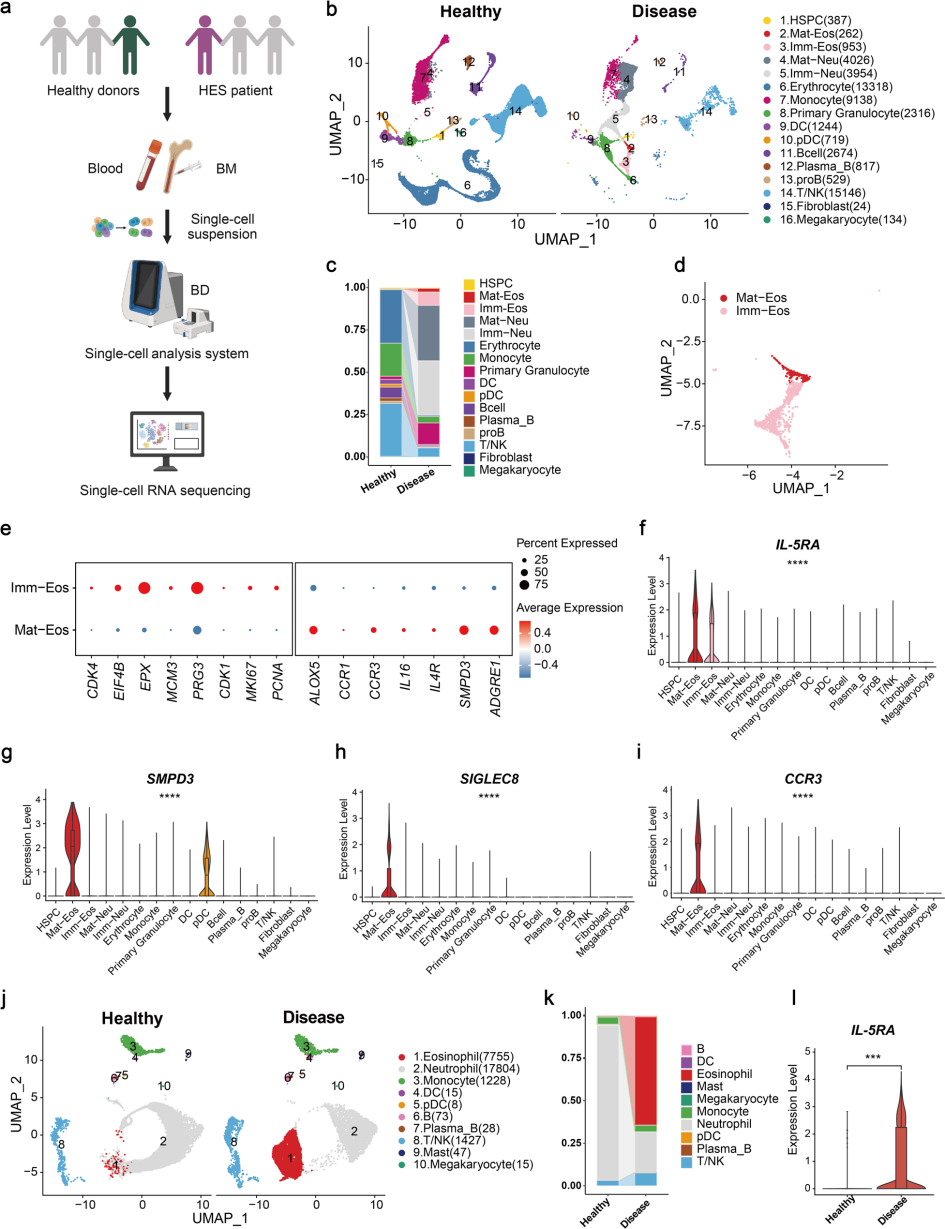
Characterization of human eosinophils in hypereosinophilic syndrome at single cell resolution. **a** Schematic overview of the experimental design and bioinformatics flow. **b** UMAP plot showing all cell subpopulations in bone marrow from healthy volunteers and a patient with hypereosinophilic syndrome (HES). Values in parentheses indicate the number of cells for each cell type. **c** Histogram of cell type proportions across bone marrow samples. **d** UMAP plot showing two subpopulations of isolated eosinophils: mature-eosinophil (Mat-Eos) and immature-eosinophil (Imm-Eos) in bone marrow from healthy volunteers and a patient with HES. **e** Dot plot visualizing the expression patterns of canonical marker genes in mature and immature eosinophil subsets derived from patient bone marrow. Color represents the normalized value of gene expression, and dot size indicates the percentage of cells expressing the gene within each cell type. **f-i** Violin plots of IL-5RA (**f**), SMPD3 (**g**), SIGLEC8 (**h**), and CCR3 (**i**) expression in bone marrow cell subpopulations from an HES patient.** j** UMAP plot showing all peripheral blood cell subpopulations from one healthy volunteer and one patient with HES. Values in parentheses indicate the number of cells for each cell type. **k** Histogram of cell type proportions across peripheral blood samples. **l** Violin plot showing IL-5RA expression across peripheral blood eosinophil subtypes from one healthy volunteer and one patient with HES. Statistical significance in (**f-i**, **l**) was assessed using the Wilcoxon rank-sum test. ns, not significant; *P < 0.05, **P < 0.01, ***P < 0.001, ****P < 0.0001. Elements in **a **were created using BioRender.com.

Comprehensive single-cell RNA sequencing (scRNA-seq) analysis of peripheral blood (PB) and bone marrow (BM) from healthy donors and a patient with HES delineated the pathogenic cellular architecture underlying disease, providing a direct rationale for therapeutic target selection (Fig. 1a). Analysis revealed a striking expansion of both mature eosinophils (Mat-Eos) and immature eosinophils (Imm-Eos) within the BM of the HES patient compared to healthy controls (Fig. 1b-d, S1a). This expansion indicates that the BM serves as a critical site of pathological eosinophil production in HES, highlighting the need for therapeutic strategies capable of targeting eosinophil populations across all developmental stages, specifically within the BM (the pathological production reservoir) and the PB (the site of pathological accumulation and effector function).

Stage-specific marker expression analysis revealed distinct profiles between subsets: Imm-Eos exhibited high expression of progenitor-/proliferation-associated markers (e.g., MKI67, PCNA), while Mat-Eos demonstrated elevated levels of lineage-specific effector molecules (e.g., ALOX5, SMPD3) (Fig. 1e). Comprehensive surface receptor profiling across immune cell populations identified IL-5Rα as the optimal membrane protein, exhibiting high expression on both developmental stages of eosinophils (Fig. 1f, S1b). In contrast, SMPD3 showed predominant expression on mature eosinophils but was also significantly detected in plasmacytoid dendritic cells (pDCs) (Fig. 1g, S1b), thereby compromising its target specificity. Siglec-8 and CCR3 expression was largely restricted to mature eosinophils (Fig. 1h-i, S1b). Further scRNA-seq analysis of the pathologically expanded PB revealed dual pathogenic alterations: quantitative expansion of eosinophils in HES patient versus healthy control, and qualitative dysregulation evidenced by significantly elevated IL-5RA expression on patient-derived eosinophils (Fig. 1j-l, S1c).

Comprehensive receptor profiling identified IL-5Rα as the optimal therapeutic target for ablating pathogenic eosinophils, based on three key properties: [1] Markedly higher IL-5Rα expression in HES patient versus healthy donors underscores its disease-specific relevance; [2] Its high expression on immature eosinophils in BM (pathological production source) and mature eosinophils in PB (effector cell reservoir) enables pan-lineage targeting across eosinophil development; [3] Its specific expression profile compared to other candidates (e.g., SMPD3 with on-target pDC expression), minimizes on-target toxicity potential. This molecular understanding directly motivated the innovative design of chimeric antigen receptor T-cell (CAR-T) therapy employing human IL-5 (hIL-5) as the natural ligand within the CAR extracellular domain. The construct was specifically engineered to engage IL-5Rα and eliminate IL-5Rα-expressing eosinophils and their precursors across both BM and PB.

### Construction and manufacturing of hIL-5 CAR-T cells

**Fig. 2:**
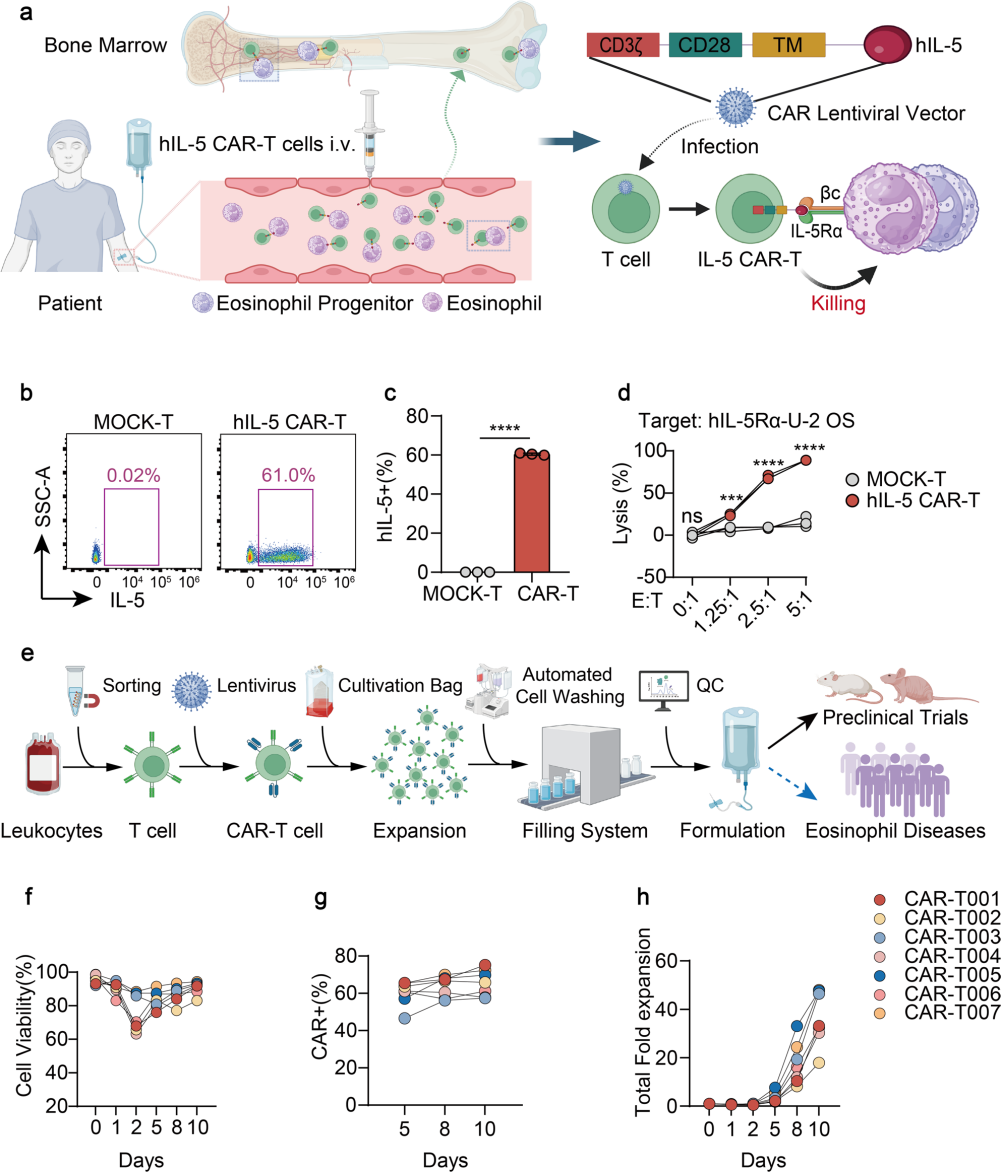
Construction and manufacturing of hIL-5 CAR-T cells.** a** Schematic of hIL-5 CAR-T cell design and mechanism of action. hIL-5 CAR-T cells are generated using a lentiviral vector encoding human IL-5 fused to CD8α hinge and transmembrane domains, followed by CD28 and CD3ζ signaling domains, and selectively target IL-5Rα-expressing eosinophils and progenitors. **b** Representative flow cytometry dot plots showing the detection of hIL-5 CAR expression on MOCK-T and hIL-5 CAR-T cells, assessed 48 h after transduction. **c** Flow cytometry analysis showing the proportion of hIL-5 CAR-positive T cells. **d** Cytotoxic activity of hIL-5 CAR-T cells against hIL-5Rα-expressing U-2 OS cells, assessed by a luciferase-based assay. **e** Schematic representation of the GMP-compliant, industrial-scale manufacturing process of hIL-5 CAR-T cells. **f** Viability of hIL-5 CAR-T cells monitored over 10 days using an automated cell counter. **g** Dynamic monitoring of hIL-5 CAR expression by flow cytometry. **h** Expansion capacity of hIL-5 CAR-T cells in vitro. CAR-T001-CAR-T007 denote seven independent manufacturing batches. All quantitative data (**c**, **d**, **f**-**h**) are shown as mean ± SD. *P* values were calculated by unpaired t-test (**c**) or two-way ANOVA (**d**); *P < 0.05, **P < 0.01, ***P < 0.001, ****P < 0.0001; ns, not significant. Elements in **a** and **e** were created using BioRender.com.

To target diseases characterized by excessive eosinophil production, such as HES and CEL, we used human IL-5 (hIL-5) as the CAR extracellular domain instead of single-chain variable fragment (scFv) to specifically eliminate hIL-5Rα-expressing eosinophils and their progenitors in both PB and BM (Fig. 2a). The construction of CAR-T cells demonstrated high efficiency, exceeding 61% (Fig. 2b and c). In vitro assays indicate that hIL-5 CAR-T cells exhibit significant and specific cytotoxic activity against U-2 OS cells overexpressing hIL-5Rα (Fig. 2d).

To comprehensively evaluate the therapeutic potential of our novel hIL-5 CAR-T cell therapy for eosinophilic malignancies, we implemented a GMP-certified manufacturing strategy. This strategy encompassed three validated critical unit operations: (1) Closed-system immunomagnetic bead selection for high-purity human CD3^+^ T cell isolation, (2) Lentiviral vector-mediated CAR gene transfer via optimized transduction protocols, and (3) Multi-parametric flow cytometry-based CAR surface expression validation. This technology transfer framework successfully met all predefined critical quality attributes (CQAs) for clinical-grade cellular products, fulfilling identity, purity, potency, and safety requirements for translational development (Fig. 2e). Viability assessments confirmed sustained cellular fitness throughout manufacturing, verifying the preservation of biological activity essential for effector functions (Fig. 2f). CAR surface expression was systematically quantified using flow cytometry with validated detection methodologies, demonstrating robust transgene expression levels critical for therapeutic functionality (Fig. 2g). Expansion potential analysis revealed substantial proliferative capacity under optimized culture conditions, achieving clinically relevant cell yields necessary for therapeutic dosing (Fig. 2h). These collective parameters validate the robustness of the manufacturing process in consistently generating functional CAR-T cell products that meet key quality benchmarks for clinical translation.

### In vitro specific cytotoxicity of hIL-5 CAR-T cells

**Fig. 3:**
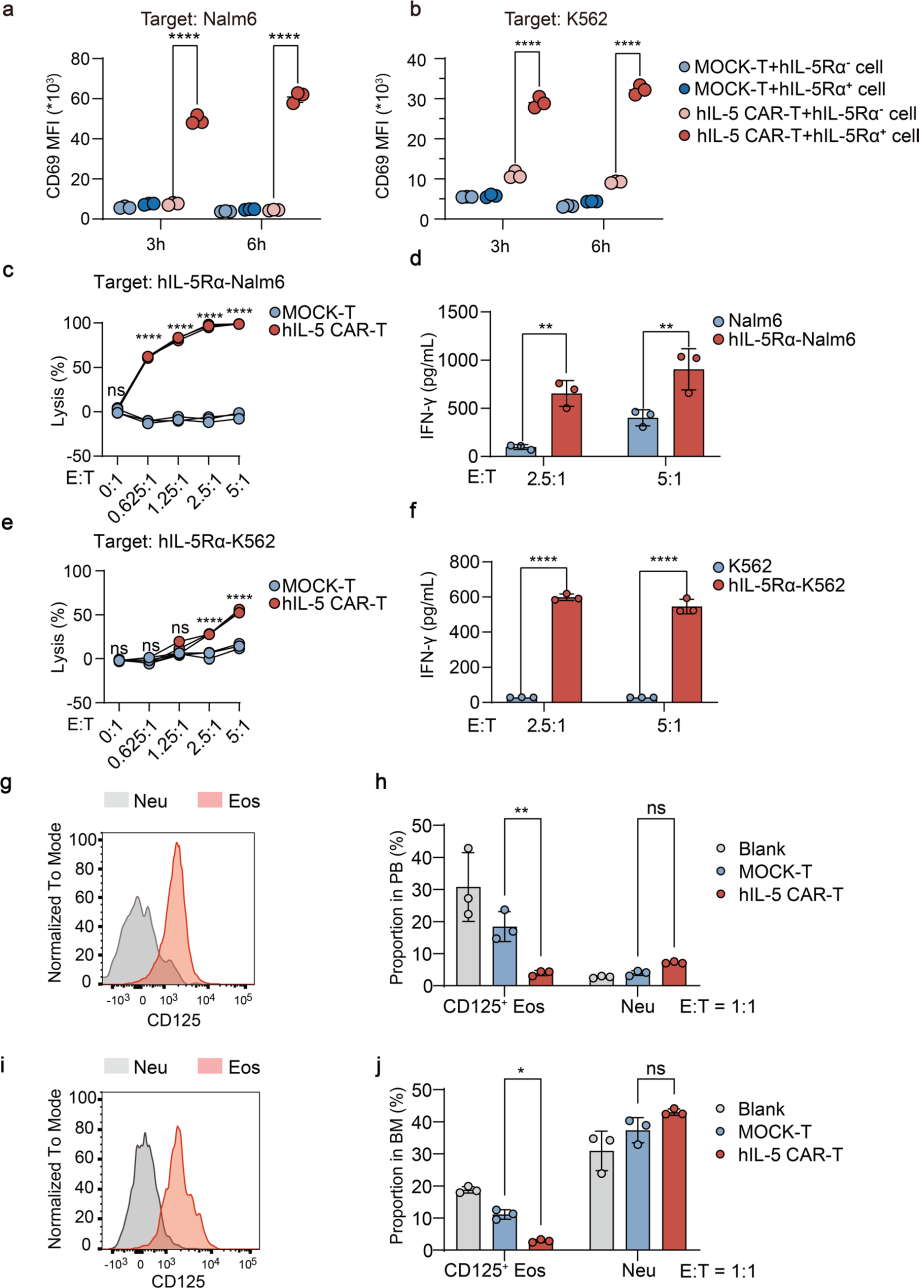
In vitro functionality evaluation of hIL-5 CAR-T cells. **a-b** Activation of CAR-T cells following co-culture with hIL-5Rα^-^ or hIL-5Rα^+^ target cells for 3 or 6 hours. CD69 expression was analyzed by flow cytometry as a marker of CAR-T cell activation. Target cell lines included Nalm6 (**a**) and K562 (**b**). **c**, **e** Cytotoxic activity of hIL-5 CAR-T cells as determined by a bioluminescence assay using luciferase-expressing hIL-5Rα^+^ target cells. Target cell lines included hIL-5Rα-Nalm6 (**c**) and hIL-5Rα-K562 (**e**). **d**, **f** The production of interferon-γ (IFN-γ) in the supernatant of hIL-5 CAR-T cells after coculture with hIL-5Rα-Nalm6 (**d**) or hIL-5Rα-K562 (**f**) target cells for 24 h was determined by ELISA. **g**,** i** Flow cytometry analysis of CD125 expression on eosinophils (Eos) and neutrophils (Neu) in peripheral blood granulocytes (**g**) and bone marrow (**i**) from a patient sample.** h**, **j **After 24-hour co-culture of patient-derived peripheral blood granulocytes (**h**) and bone marrow (**j**) with either hIL-5 CAR-T or MOCK-T cells, flow cytometry was used to determine the proportion of CD125^+^ eosinophils or neutrophils, to assess the specificity of hIL-5 CAR-T cell-mediated cytotoxicity. Data are mean ± SD. Significance was determined by two-way ANOVA (**a-f**, **h**, **j**); *P < 0.05, **P < 0.01, ***P < 0.001, ****P < 0.0001; ns, not significant.

The in vitro cytotoxicity of hIL-5 CAR-T cells was comprehensively evaluated using a co-culture system with various target cell lines. The activation of hIL-5 CAR-T cells was assessed in the presence of two different cell lines: Nalm6 and K562. The results showed that hIL-5 CAR-T cells exhibited specific activation, as evidenced by the upregulation of CD69, a T-cell activation marker, in response to hIL-5Rα-expressing target cells (Fig. 3a and b). The antigen-specific cytotoxic potential of hIL-5 CAR-T cells was profiled using a luminescent cytotoxicity assay. Upon co-culture with hIL-5Rα-expressing target cells, hIL-5 CAR-T cells efficiently induced target cell lysis in a dose-dependent manner (Fig. 3c and e). Additionally, IFN-γ release by hIL-5 CAR-T cells was measured, revealing a significant increase upon target cell engagement (Fig. 3d and f). This cytokine release indicated a robust immune response mediated by hIL-5 CAR-T cells, further supporting their potential as an effective therapeutic strategy.

Flow cytometric analysis of HES patient samples revealed dominant IL-5Rα (CD125) expression on pathological eosinophils in PB (Fig. 3g) and BM (Fig. 3i). Following co-culture with hIL-5 CAR-T cells, marked depletion of CD125 positive eosinophils population was observed in both PB (Fig. 3h) and BM (Fig. 3j). Crucially, neutrophil proportions remained fully preserved with no observable impact. Overall, the results support selective targeting of eosinophils, with non-target hematopoietic cells appearing largely preserved in the assays performed.

### Safety evaluation of hIL-5 CAR-T cells

**Fig. 4:**
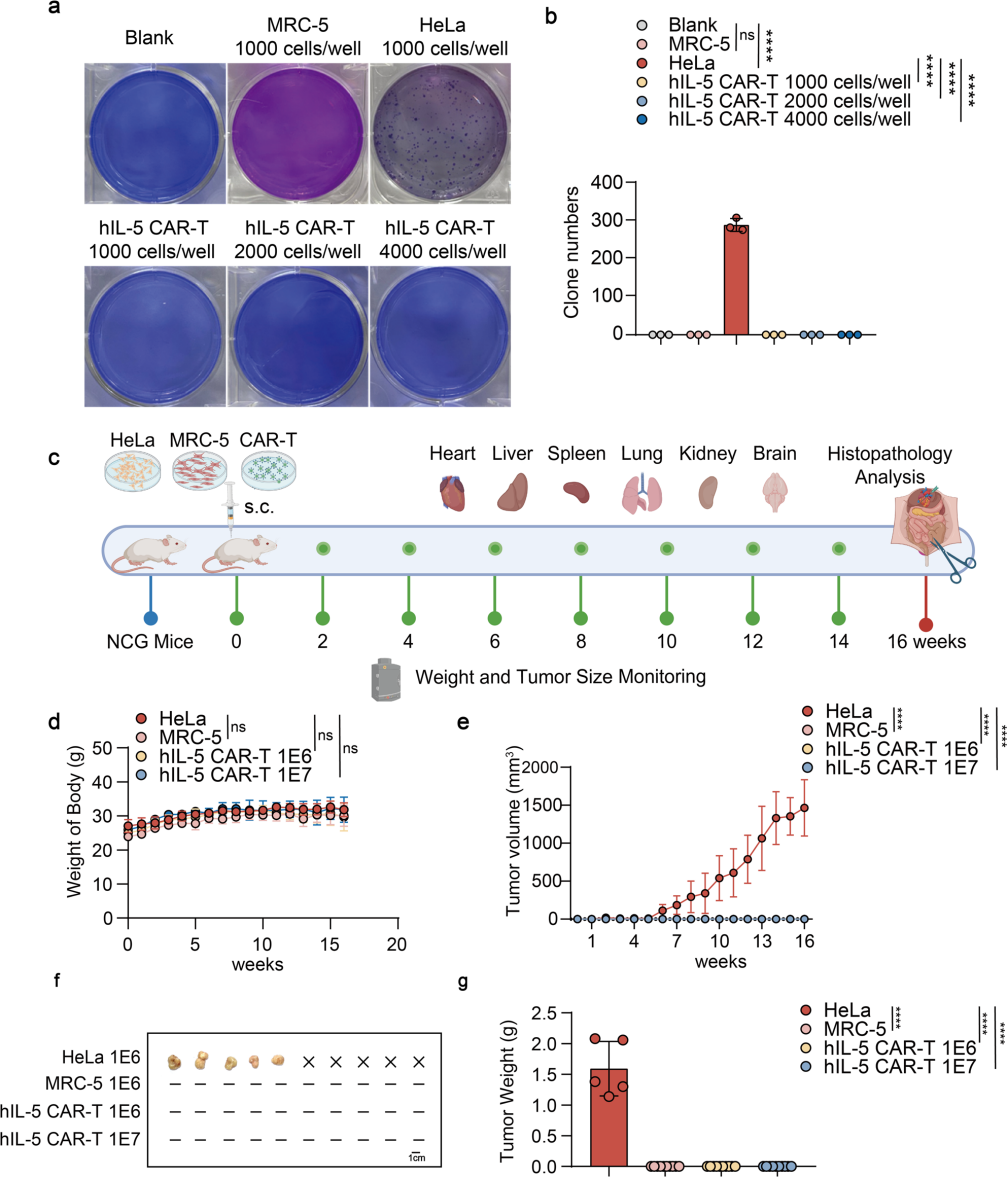
Tumorigenicity assessment of hIL-5 CAR-T cells.** a** Soft agar colony formation assay evaluating the ability of hIL-5 CAR-T cells to form colonies after two weeks of culture. **b** Quantification of colony numbers in each group from (**a**). **c** Schematic overview of the in vivo tumorigenicity study. NCG mice were subcutaneously injected with HeLa, MRC-5, 1 × 10^6^ hIL-5 CAR-T or 1 × 10^7^ hIL-5 CAR-T cells (*n =* 10). **d** Body weight changes of mice during the observation period. **e** Tumor volume measurements over time during the study. **f** Tumors harvested from mice at the ethical endpoint. “×” indicates mice euthanized upon reaching ethical tumor endpoints;  “–”  indicates no tumor development during the observation period. **g** Tumor weight measurements corresponding to (**f**). All graphs show mean ± SD. Statistical significance was determined by one-way ANOVA (**b**, **g**) and two-way ANOVA (**d**, **e**). *P < 0.05, **P < 0.01, ***P < 0.001, ****P < 0.0001; ns, not significant. Elements in **c** were created using BioRender.com.

To bridge preclinical discovery with therapeutic applications, the tumorigenic potential and systemic toxicity of hIL-5 CAR-T cells were rigorously assessed through complementary in vitro and in vivo models. Soft agar colony formation assay confirmed the absence of spontaneous tumorigenicity under controlled conditions (Fig. 4a and b). In subcutaneously injected NCG mice (Fig. 4c-g) and nude mice (Fig. S2, S3, S4), monitoring demonstrated the absence of ectopic tumorigenesis and maintained body weight homeostasis (Fig. 4c-g, S2). This was corroborated by H&E-stained organ sections, which showed preserved tissue architecture without pathological changes (Fig. S3). Immunohistochemical staining for human CD3 and IL-5 in major organs showed minimal to no detectable signal, indicating that following subcutaneous administration, CAR-T cells did not migrate to or infiltrate peripheral tissues or organs, thereby further confirming the lack of tumorigenic potential (Fig. S4).

**Fig. 5:**
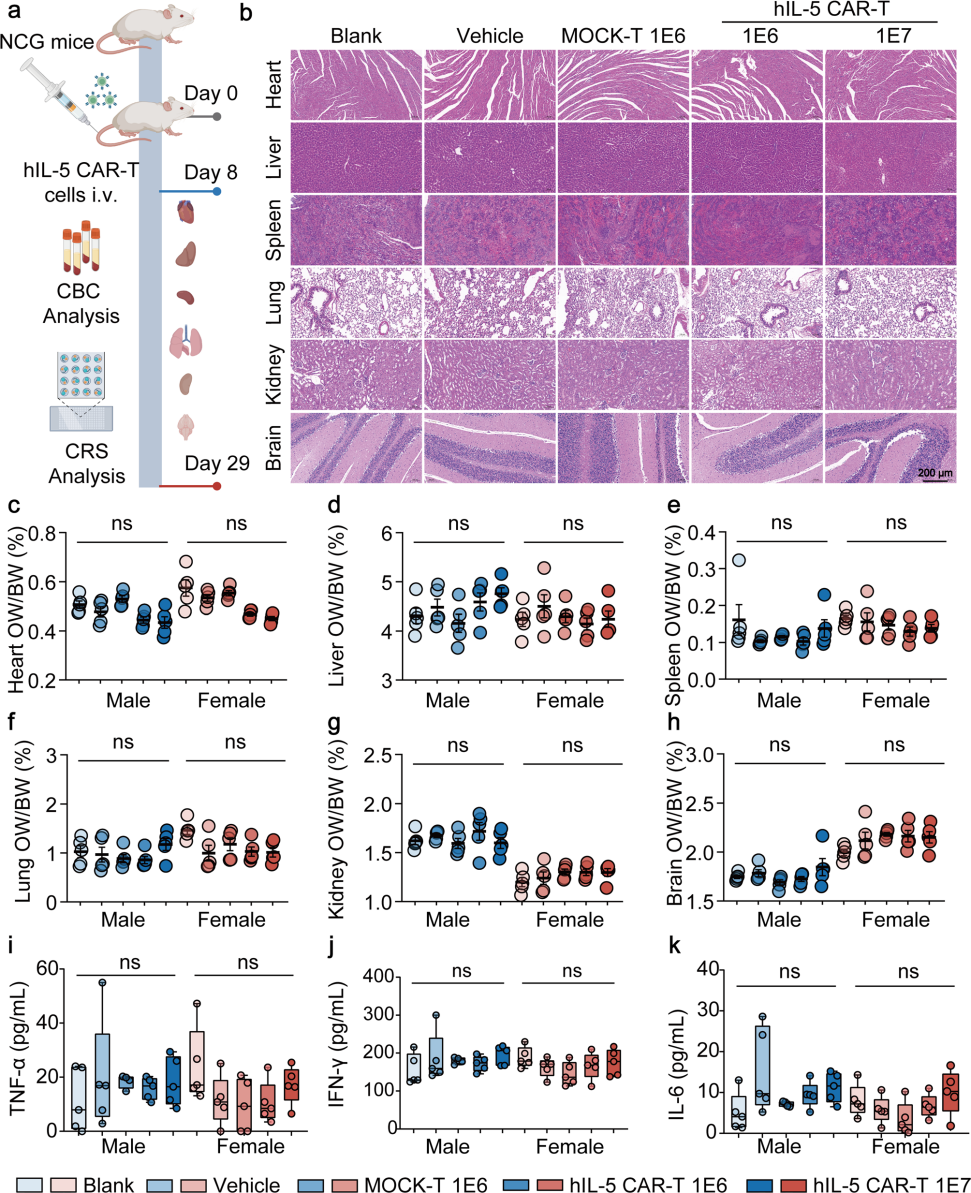
In vivo toxicity assessment of hIL-5 CAR-T cells. **a** Schematic overview of the in vivo toxicology study. NCG mice were divided into five groups: blank control, vehicle control, 1×10^6^ MOCK-T cells, 1×10^6^ hIL-5 CAR-T cells, and 1×10^7^ hIL-5 CAR-T cells (*n*=10). Cells were administered intravenously (i.v.). Systemic toxicity, organ toxicity, complete blood count (CBC) and cytokine release syndrome (CRS) were conducted on day 8 and day 29 post-infusion. **b** Representative H&E staining of major organs collected on day 29 post-CAR-T infusion. Scale bars, 200 μm. **c-h** Organ-to-body weight ratios of the heart (**c**), liver (**d**), spleen (**e**), lung (**f**), kidney (**g**) and brain (**h**) measured on day 29 post-CAR-T infusion. **i-k** Human serum cytokine levels of TNF-α (**i**), IFN-γ (**j**) and IL-6 (**k**), measured on day 8 post-CAR-T infusion using the RayPlex® Human Inflammation Array Kit. Data in panels ( **c-h**) are presented as mean ± SEM, and in panels (**i-k**) are presented as mean ± SD. Statistical significance was determined by two-way ANOVA (**c-h**) or one-way ANOVA (**i-k**). *P < 0.05, **P < 0.01, ***P < 0.001, ****P < 0.0001; ns, not significant. Elements in **a** were created using BioRender.com.

To further assess systemic safety, we administered hIL-5 CAR-T cells to NCG mice and evaluated the histoarchitectural integrity of major organ systems. No evidence of inflammatory infiltration, necrosis, or fibrosis was observed, and organ-to-body weight ratios remained within normal physiological ranges (Fig. 5a-h, S5-S9). Macroscopic evaluation showed no local reactions (inflammation) at injection sites compared to controls (Fig. S10). Cytokine profiling revealed that key proinflammatory cytokines associated with cytokine release syndrome (CRS), such as IL-6, IFN-γ, and TNF-α, remained within physiological thresholds, indicating stringent maintenance of cytokine homeostasis (Fig. 5i-k, S11). Hematological parameters (WBC differentials, erythrocyte indices) showed no clinically relevant deviations (Fig. S12 and S13).

Additionally, to assess the potential safety risks in the tumor microenvironment, we established an NCG mouse model engrafted with hIL-5Rα-expressing Nalm6 cells, followed by systemic administration of the CAR-T cells (Fig. S14a). Histopathological analysis of major organs in all experimental groups confirmed the absence of detectable toxicity, with no evidence of inflammation, necrosis, or fibrosis (Fig. S14b). CAR-T cells exhibited stable physiological conditions. Core body temperature remained within the normal range (35–38 °C) throughout a 60-minute monitoring period, showing no significant fluctuations relative to the blank, tumor, or MOCK-T control groups (Fig. S14c). Furthermore, PB analysis indicated stable levels of histamine (Fig. S14d) and no significant shifts in the proportions of major immune cell subsets (Fig. S14 e-g). PB cytokine profiling showed no significant elevation of CRS-associated mediators in the hIL-5 CAR-T group compared to controls, further validating the therapeutic safety of this CAR-T cell treatment (Fig. S14 h-t).

CyTOF analysis of immune cells from healthy human peripheral blood confirmed that only eosinophils exhibited high IL-5Rα expression, whereas other immune subsets showed either undetectable or weak expression (Fig. S15). In mice, IL-5Rα expression was also low on basophils, compared to eosinophils (Fig. S16).

Collectively, these data demonstrated that hIL-5 CAR-T cells exhibit a favorable safety profile, with no tumorigenicity, cytokine-associated toxicities, or anaphylactic reaction. The demonstrated target specificity and absence of tissue cytotoxicity support clinical development for eosinophilic disorders.

### Anti-tumor efficacy of hIL-5 CAR-T cells in models of hypereosinophilic leukemia

**Fig. 6:**
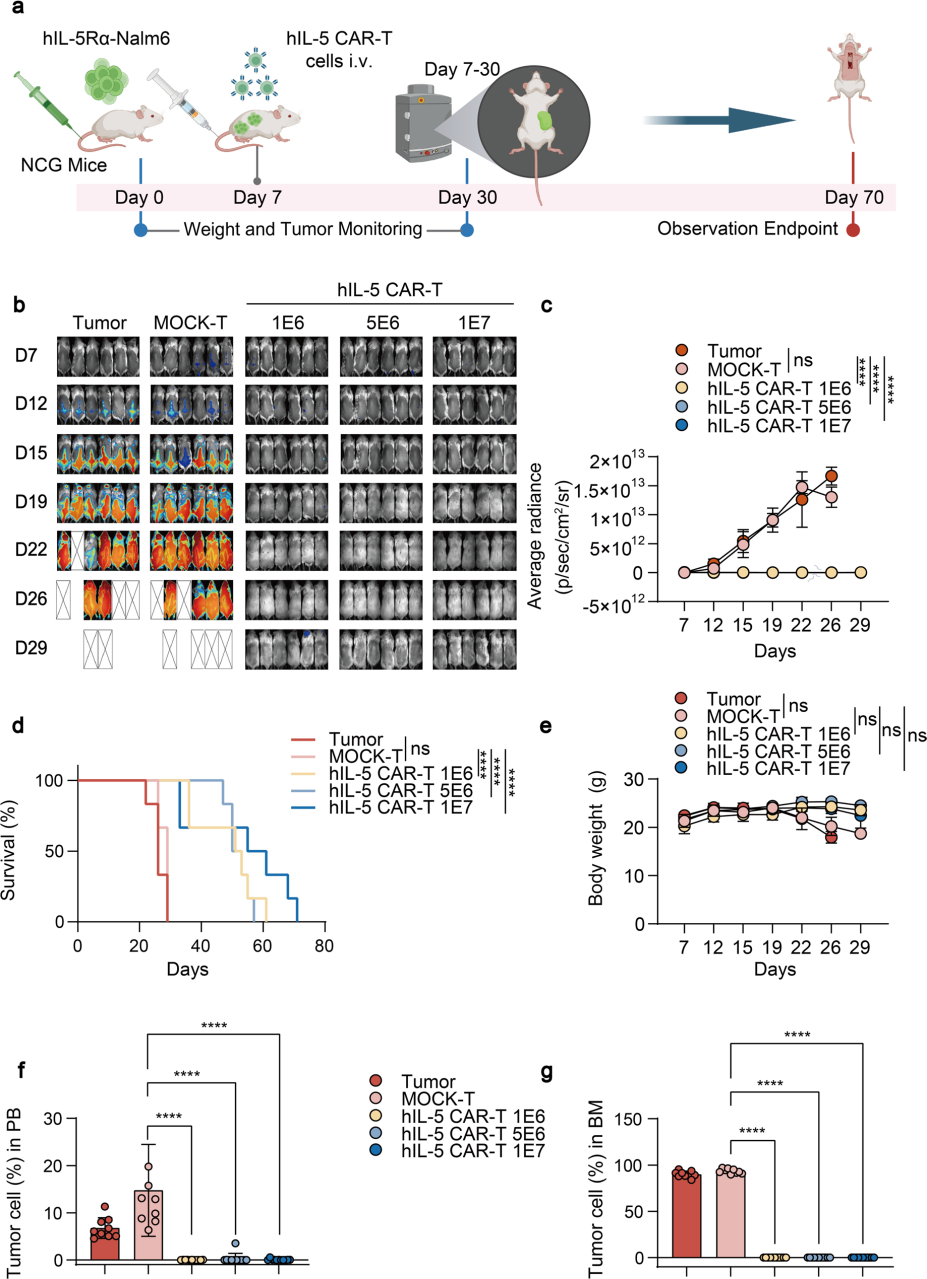
Anti-tumor efficacy of hIL-5 CAR-T cells in a hypereosinophilic leukemia model. **a** Schematic of the in vivo antitumor efficacy study. NCG mice were intravenously injected with hIL-5Rα-Nalm6 cells and treated on day 7 with MOCK-T (1 × 10^6^) or escalating doses of hIL-5 CAR-T cells (1 × 10^6^, 5 × 10^6^, or 1 × 10^7^; *n* = 6). Tumor burden was assessed by bioluminescence imaging, along with body weight and survival. **b** Bioluminescence imaging of tumor burden at the indicated time points following CAR-T cell infusion. “×” indicates mice reaching the ethical endpoint. **c** Quantification of leukemia burden based on average bioluminescence intensity from (**b**) (*n *= 6). **d** Kaplan-Meier survival curves depicting overall survival. **e** Body weight changes of mice during the study. **f** Proportion of tumor cells in peripheral blood at day 26. **g** Proportion of tumor cells in mouse bone marrow at termination on day 26. Statistical significance was determined by two-way ANOVA (**c-g**) or log-rank (Mantel-Cox) test (**d**). *P < 0.05, **P < 0.01, ***P < 0.001, ****P  <0.0001; ns, not significant. Elements in **a** were created using BioRender.com.

To evaluate the anti-tumor efficacy of hIL-5 CAR-T cells, we first utilized a NCG mouse model of hypereosinophilic leukemia, employing Nalm6 cells expressing IL-5Rα. The protocol involved adoptive transfer of genetically engineered T cells into tumor-bearing hosts. In vivo bioluminescence imaging was deployed to non-invasively quantify tumor dynamics following CAR-T infusion (Fig. 6a). Bioluminescence imaging demonstrated that the hIL-5 CAR-T treated groups, including the lowest-dose cohort, exhibited a reduction in bioluminescent signals compared to the MOCK-T controls, with statistical significance (Fig. 6b and c). Survival analysis revealed that hIL-5 CAR-T cells conferred significant therapeutic benefits in hIL-5Rα-Nalm6-bearing NCG mice, with the high-dose cohort exhibiting more than a twofold extension in median survival time compared to MOCK-T controls (Fig. 6d). It must be noted, consistent with the challenges observed in other highly aggressive xenograft models [30], all mice in the CAR-T cell treatment groups ultimately succumbed to tumor relapse, indicating that while treatment markedly prolonged survival, it did not achieve a complete cure in this specific aggressive model (Fig. S17).

These results underscore the precision and potency of hIL-5 CAR-T cells in eradicating malignant eosinophilic cells. Throughout the therapeutic intervention, body weight remained stable, confirming a favorable safety profile (Fig. 6e). Quantification of tumor cells in PB and BM revealed that MOCK-T group exhibited aggressive leukemic infiltration. In contrast, hIL-5 CAR-T treatment significantly reduced tumor burden (Fig. 6f and g). Combined gross examination and organ weight analysis confirmed resolution of leukemic splenomegaly following hIL-5 CAR-T cell therapy (Fig. S18a and b). Flow cytometric quantification demonstrated that hIL-5 CAR-T cell therapy significantly reduced splenic tumor burden across all doses (Fig. S18c and d).

In the K562-based hypereosinophilic leukemia model (Fig. S19a), hIL-5 CAR-T or MOCK-T cells were administered on Day 7, bioluminescence imaging showed a marked reduction in tumor fluorescence in the hIL-5 CAR-T treated groups (Fig. S19 b and c). Survival analysis revealed an extension in median survival time for the hIL-5 CAR-T group compared to MOCK-T controls (Fig. S19d), with no significant differences in body weight between the groups, indicating a favorable safety profile for the hIL-5 CAR-T treatment (Fig. S19e).

In an IL-33 alarmin-induced eosinophilia model [8] (Fig. S20a and e), both mIL-5 CAR-T cells administered five days before modeling (Day − 5) and on the day of modeling (Day 0) significantly reduced eosinophil counts in the BM (Fig. S20b and f), PB (Fig. S20c and g), and spleen (Fig. S20d and h) compared to the MOCK-T group, further supporting the therapeutic potential of CAR-T treatment in eosinophil-driven diseases.

These experimental data demonstrated that IL-5 CAR-T cells effectively eliminate eosinophils in both the humanized NCG mouse models of hIL-5Rα-positive leukemia and the IL-33 alarmin-induced eosinophilia model, supporting the therapeutic potential of hIL-5 CAR-T cells in eosinophil-driven diseases.

## Discussion

Our study represents a significant advancement in CAR-T cell therapy by pioneering the development of hIL-5 CAR-T cells that specifically target eosinophils in HES and CEL. This innovative approach broadens the therapeutic scope of CAR-T cell therapy, providing a targeted solution to controlling eosinophil-driven disease processes and tissue damage.

Monoclonal antibodies (mAbs) targeting the IL-5/IL-5Rα pathway, such as mepolizumab and benralizumab, have proven effective in treating HES [11, 12]. However, a subset of patients experience incomplete responses, relapse upon treatment discontinuation, or persistent disease driven by eosinophil progenitors [12, 31, 32]. Unlike mAbs, which require repeated dosing, CAR-T cells offer the potential for long-term persistence, with a single infusion potentially leading to durable remission [33]. This makes CAR-T therapy a promising alternative, particularly for patients with refractory or relapsed disease. Chen et al. were the first to validate the therapeutic potential of IL-5 CAR-T cells, demonstrating their ability to effectively deplete eosinophils in asthma models [34]. Similarly, Jin et al. further confirmed the long-term efficacy of engineered CAR-T cells targeting eosinophils in type 2 inflammatory diseases [35]. This study extends the application of IL-5 CAR-T cell therapy from asthma to hematologic eosinophilic diseases, integrates patient-derived expression data, and progresses from basic efficacy validation to clinical translation, supporting the feasibility of using clinical-grade humanized products to treat a broader range of eosinophil-associated diseases. Future studies should involve comparisons of hIL-5 CAR-T therapy with existing anti-IL-5/IL-5Rα monoclonal antibodies in preclinical models to directly assess their relative advantages and risks concerning durability of response, ability to overcome resistance, and long-term disease control.

Notably, hIL-5 CAR-T cells were designed to specifically target eosinophils and their progenitors, based on the predominant expression of IL-5Rα on these cells. Although low-level expression of IL-5Rα has been reported on other immune cells, such as activated B cells, the IL-5 CAR-T cells did not exhibit cytotoxicity against these B cells [35]. Our analysis of immune cells from healthy human peripheral blood confirmed that only eosinophils exhibited high IL-5Rα expression, whereas other immune subsets showed either undetectable or weak expression. We also detected low-level expression of IL-5Rα on mouse basophils. While hIL-5 CAR-T cells could theoretically target these non-eosinophil cell populations, the risk of non-specific effects on the patient is influenced by factors such as the receptor expression levels and the functional relevance of these cells. Consistent with this, our in vivo tumorigenicity and toxicology studies did not identify adverse effects attributable to hIL-5 CAR-T cells. Together, these findings suggest a favorable preclinical safety profile under the conditions tested, while further evaluation will be required in clinical settings.

Despite its promising therapeutic potential, our study also highlights several limitations that will need to be addressed in future research to better assess the safety and efficacy of hIL-5 CAR-T cell therapy. In the management of HES, a disorder characterized by sustained elevation of eosinophil counts (> 1.5 × 10^9^/L) persisting for over six months, often accompanied by end-organ damage, improving the survival and functional persistence of CAR-T cells has become a critical therapeutic focus [36]. This biological enhancement is particularly crucial for developing durable treatment strategies that could reduce the current clinical reliance on repeated therapeutic administrations and conditioning regimens. To enhance CAR-T cell persistence and functionality, multiple strategies have been developed and demonstrated promise in clinical settings, including incorporating co-stimulatory domains (e.g., CD28 or 4-1BB) and engineering T cells to express cytokines such as IL-7, IL-15, and IL-21 [37–40]. Additionally, gene editing tools like CRISPR/Cas9 can be employed to precisely knock out genes associated with T cell exhaustion and apoptosis or to overexpress anti-apoptotic genes like *Bcl-2* [41], thereby preventing T cell death and improving persistence. Furthermore, the combined application of immune checkpoint inhibitors (e.g., PD-1 [42, 43], CTLA-4 [44, 45], PTP1B [46], TIM-3 [47]) with strategies to modulate the immunosuppressive microenvironment (e.g., through TGF-β [48] or IDO [49, 50] inhibitors) has shown potential for synergistic effects leading to enhanced clinical outcomes.

While preclinical efficacy trials have garnered significant attention, balancing efficacy and safety is essential for maximizing patient benefits. Strategies such as adapter-CAR-T cells activated by small molecules or protein drugs have been developed to enhance safety profiles during treatment [51, 52]. In the production process, non-viral site-specific integration and automated platforms with higher release standards can minimize viral residues, ensuring the phenotype, purity, and stability of CAR-T cells, and reducing the risk of pathogenic contamination [53]. Within this increasingly robust safety framework, hIL-5 CAR-T cell therapy shows considerable promise for patients with chronic eosinophilic leukemia accompanied by end-organ damage and for those with tyrosine kinase inhibitor (TKI)-resistant myeloid/lymphoid neoplasms with eosinophilia and tyrosine kinase gene fusions (M/LN-eo-TK). Nevertheless, its clinical implementation should rely on careful patient selection and proactive risk management strategies to maximize therapeutic benefit while minimizing potential adverse events.

The application of IL-5 CAR-T cell therapy in treating hypereosinophilic disorders has shown promise as a potential approach for targeting eosinophils. Given the extensive physiological roles of eosinophils and their involvement in various diseases, IL-5 CAR-T cell therapy could be explored for the treatment of other eosinophil-related disorders, including asthma, atopic dermatitis [54], eosinophilic gastrointestinal disorders (EGID) [55], eosinophilic granulomatosis with polyangiitis (EGPA) [56], and bullous pemphigoid [57, 58]. Preclinical studies on the efficacy and safety of IL-5 CAR-T cells in these disease models will be critical to advancing their clinical translation. However, given the physiological roles of eosinophils, the long-term consequences of sustained eosinophil depletion will require careful evaluation in each disease context.

Although our in vivo models offer valuable proof-of-concept support for IL-5 CAR-T cell targeting, we acknowledge that they do not fully replicate the complex biology of eosinophilic malignancies like CEL and HES. Therefore, future research will focus on developing in vitro and in vivo models that more closely replicate the clinical-pathological features of CEL and HES. This will include incorporating the expression profile of key targets such as IL-5Rα, and simulating critical disease features, including tissue homing, tumor invasiveness, and eosinophil-specific cytokine expression. Moreover, patient-derived primary cell and organoid models will be employed to better preserve the tumor microenvironment and cellular heterogeneity. These approaches aim to more accurately evaluate the efficacy, persistence, and safety of IL-5 CAR-T cells under conditions that closely mirror the disease state, providing substantial data to support clinical translation.

In conclusion, our study highlights the potential of hIL-5 CAR-T cell therapy as a novel treatment for HES and CEL. By targeting eosinophils and their progenitors, this approach offers a promising alternative to current therapies. These findings also suggest the broader applicability of CAR-T technology in eosinophil-driven diseases, warranting further clinical validation to establish its safety and efficacy as a new therapeutic option for patients with refractory conditions.

## Supplementary Information

Supplementary Material 1.

## Data Availability

The human dataset is available at the Genome Sequence Archive (GSA) in China National Center for Bioinformation under accession number PRJCA055127. Any additional information required to reanalyze the data reported in this study is available from the lead contact upon request.
