# Supplementary material for: IL-5 CAR-T cell therapy induces effective remission in hypereosinophilic disorders
Source: J Hematol Oncol. 2026 Feb 13;19:18. doi: 10.1186/s13045-026-01782-x (PMC13005461; doi:10.1186/s13045-026-01782-x)
Supplement: Supplementary file 1 — Supplementary Material 1. [file 13045_2026_1782_MOESM2_ESM.pdf]

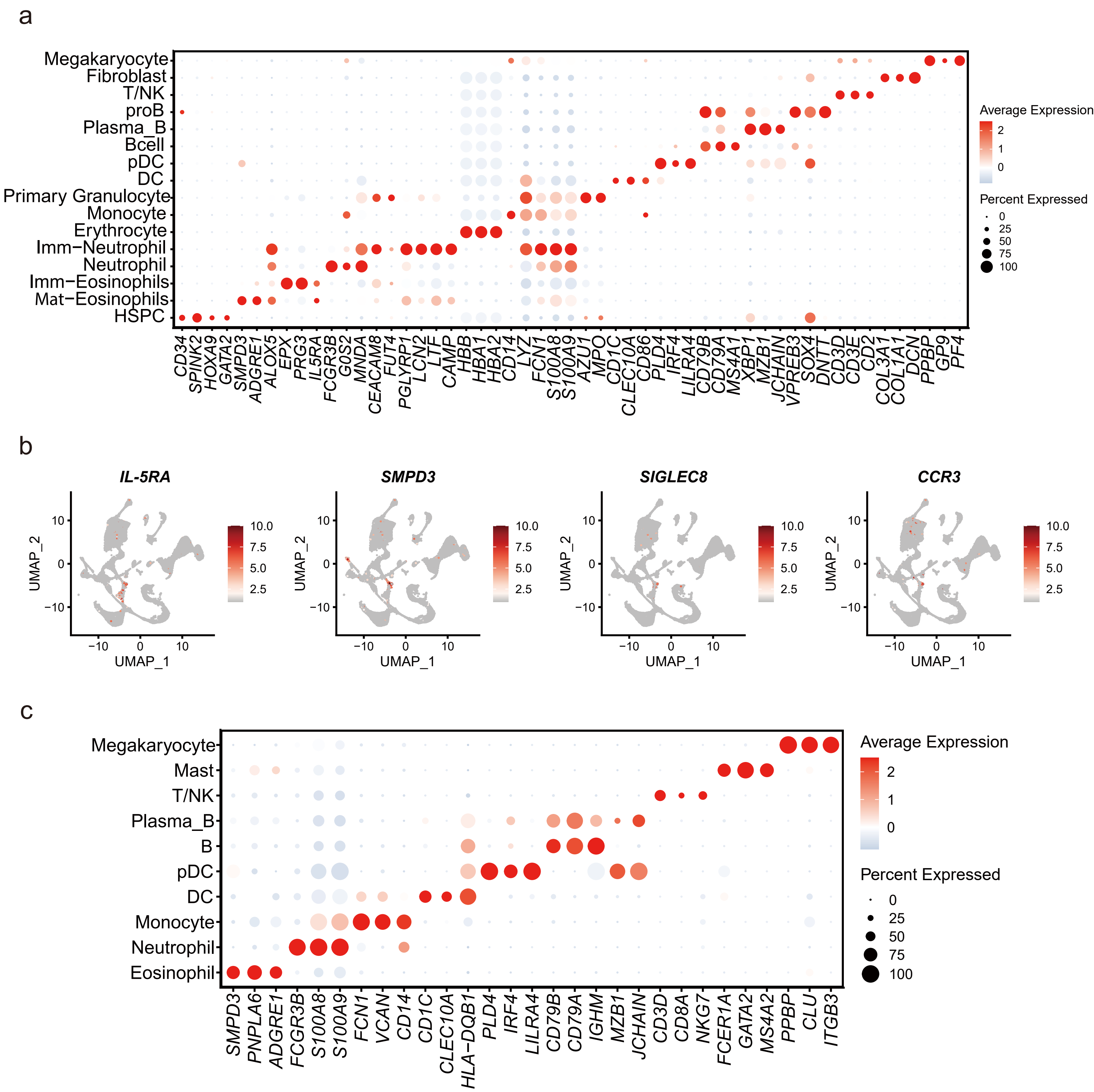

Suppl Fig1. Characterisation of human eosinophils in hypereosinophilic disorders at single cell resolution. a,c Dot plot of classical marker gene expression for different cell types. Color represents the normalized value of gene expression, and dot size indicates the percentage of cells expressing the gene within each cell type. **b** UMAP plots showing the expression distribution of *IL-5RA*, *SMPD3*, *SIGLEC8*, and *CCR3* across cell subpopulations, based on the same dataset presented in Figure **1b**.

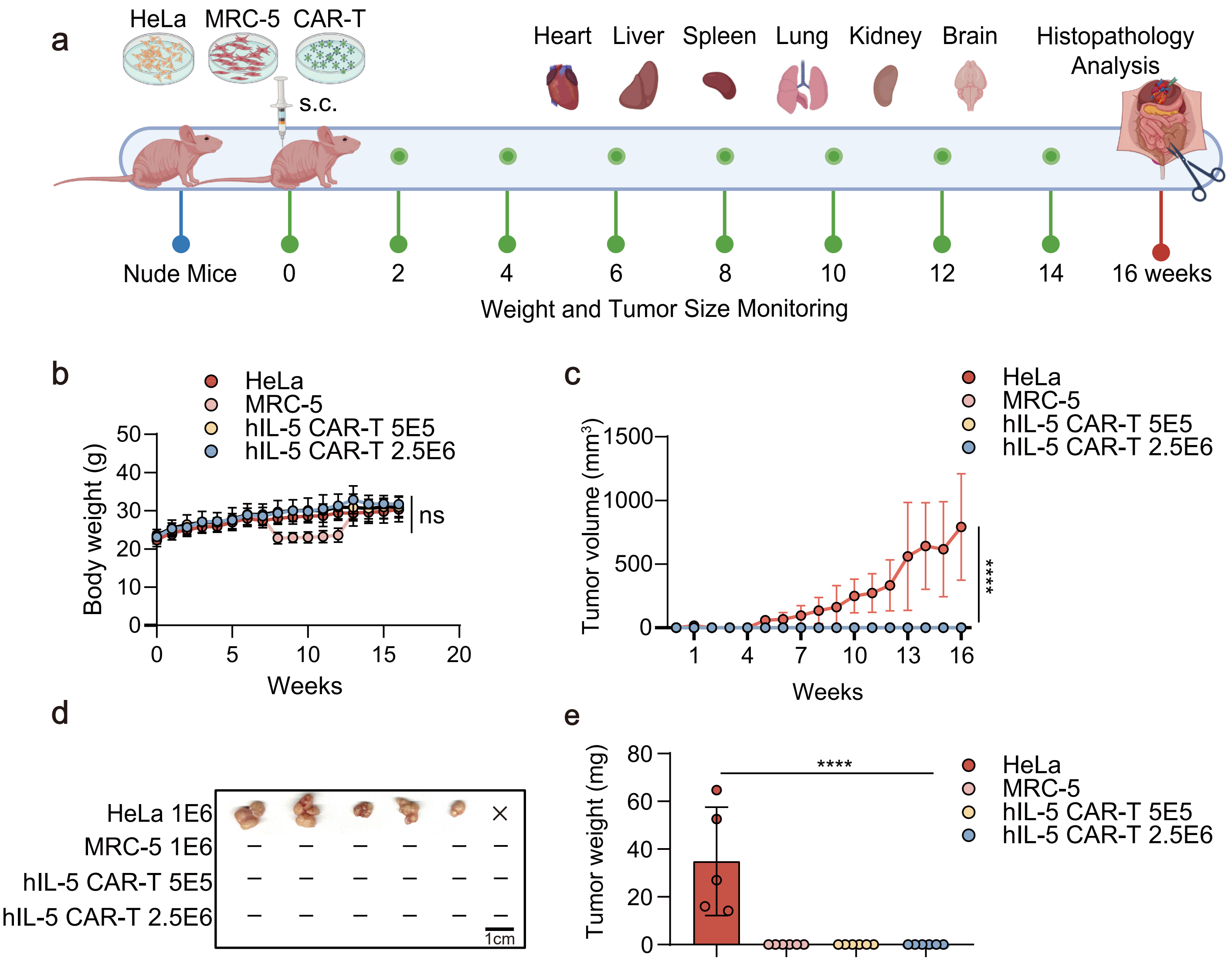

Suppl Fig2. Tumorigenicity evaluation of hIL-5 CAR-T cells. a Schematic representation of the in vivo tumorigenicity study. BALB/c nude mice were subcutaneously injected with HeLa cells, MRC-5 cells, or hIL-5 CAR-T cells at doses of 5×10^5 or 2.5×10^6 cells per mouse (n=6). Elements were created using BioRender.com. **b** Body weight curves of mice from (**a**). **c** Tumor volume measurements over time in mice from (**a**). **d** Tumors excised from mice at the experimental endpoint; "×" indicates mice euthanized upon reaching ethical tumor endpoints; "–" indicates no tumor development during the observation period. **e** Tumor weight measurements corresponding to (**d**). Data are presented as mean ± SD. *$P < 0.05$, **$P < 0.01$, ***$P < 0.001$, ****$P < 0.0001$; ns, not significant. Statistical significance was determined by two-way ANOVA (**b**,**c**) and one-way ANOVA (**e**).

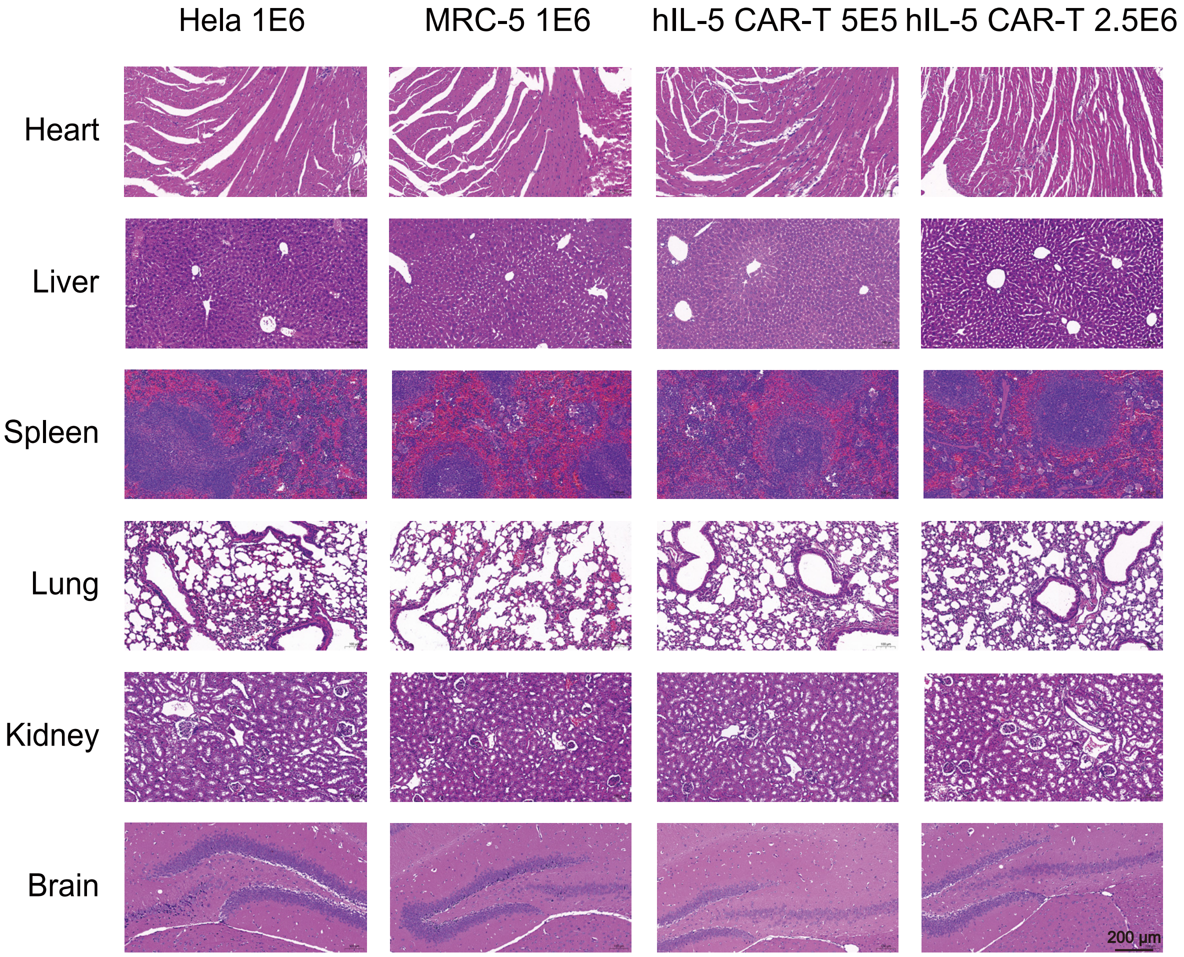

Suppl Fig3. Histological analysis of organs in the tumorigenicity assay. Representative hematoxylin and eosin (H&E) staining was performed on organs (heart, liver, spleen, lung, kidney, and brain) obtained from mice in the tumorigenicity assay. Scale bars, 200 μm.

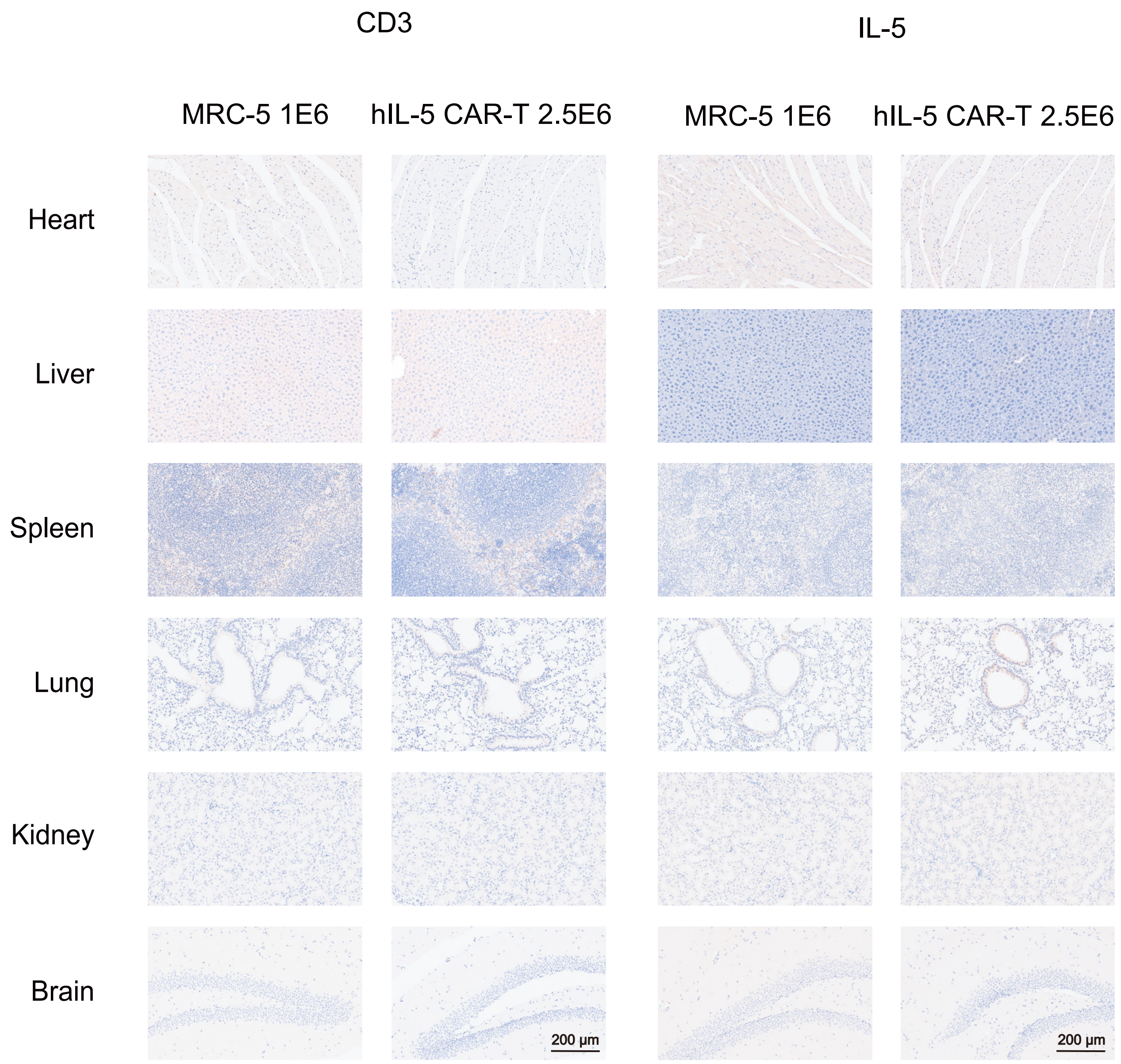

Suppl Fig4. CD3 and IL-5 IHC staining in organs in the tumorigenicity evaluation. Representative images of CD3 and IL-5 immunohistochemistry (IHC) staining in various organs in the tumorigenicity evaluation. Scale bar, 200 μm.

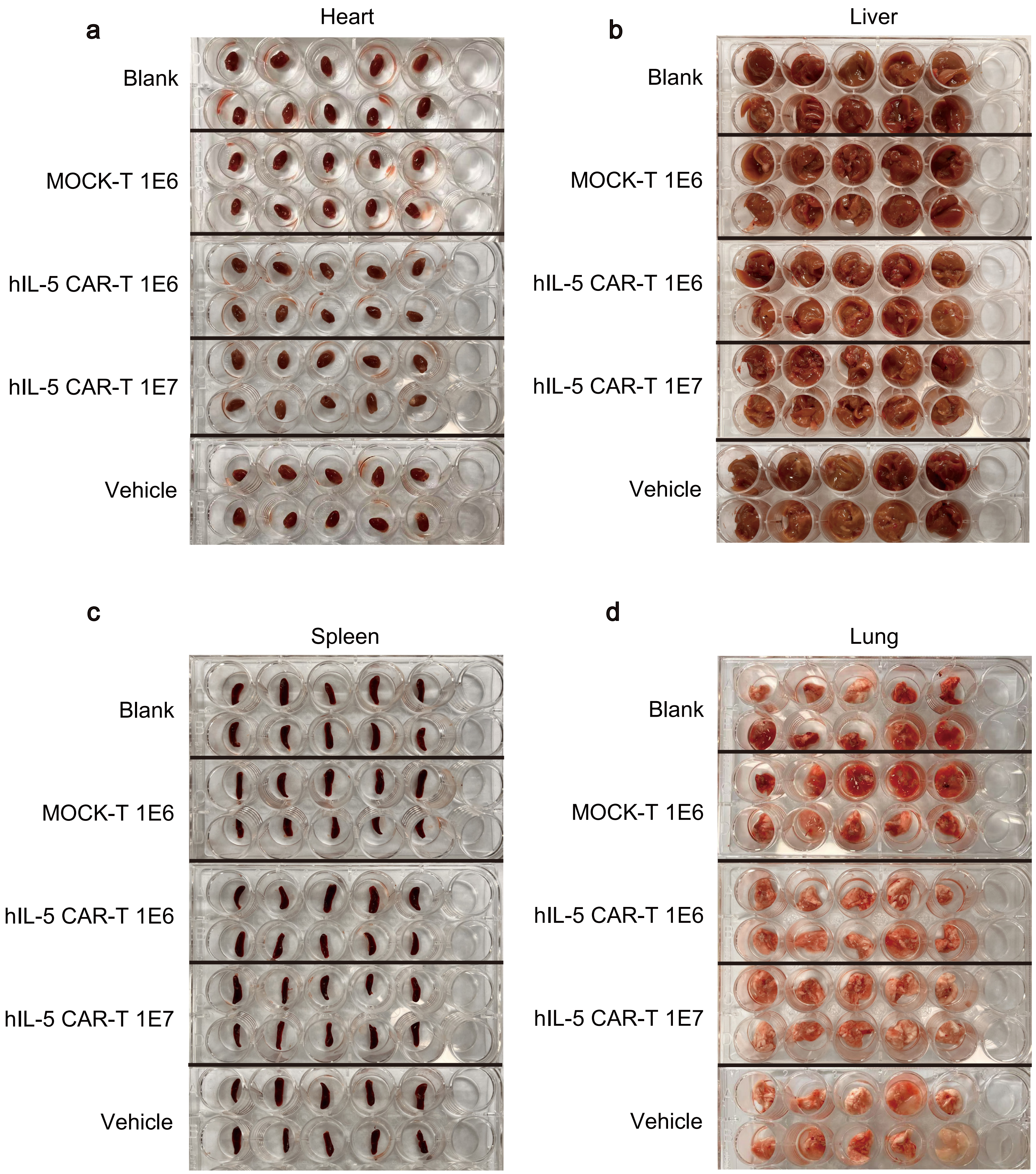

a
Heart
Blank
MOCK-T 1E6
hIL-5 CAR-T 1E6
hIL-5 CAR-T 1E7
Vehicle
b
Liver
Blank
MOCK-T 1E6
hIL-5 CAR-T 1E6
hIL-5 CAR-T 1E7
Vehicle
c
Spleen
Blank
MOCK-T 1E6
hIL-5 CAR-T 1E6
hIL-5 CAR-T 1E7
Vehicle
d
Lung
Blank
MOCK-T 1E6
hIL-5 CAR-T 1E6
hIL-5 CAR-T 1E7
Vehicle

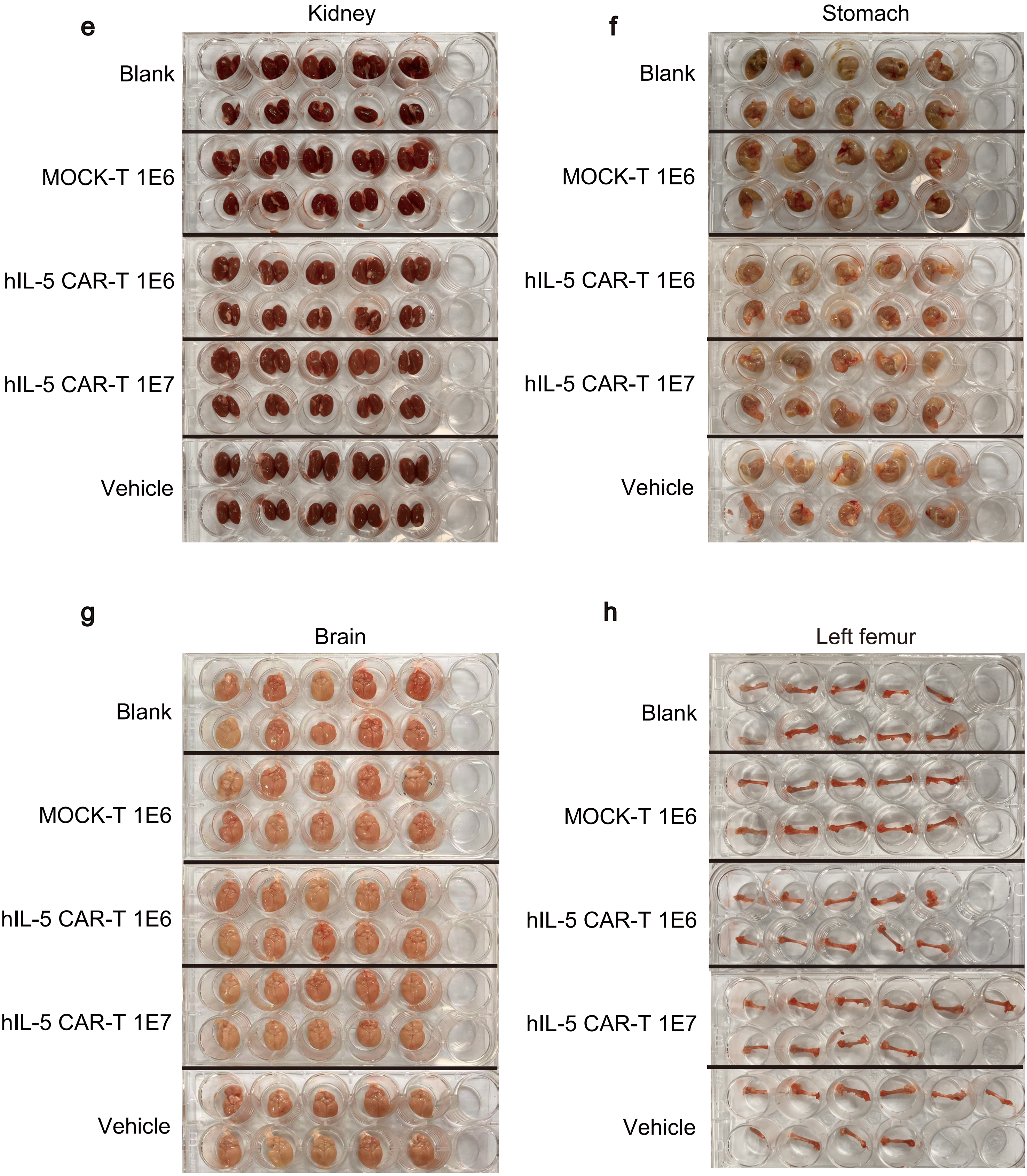

Suppl Fig5. Morphological analysis of collected organs on day 8 post-CAR-T infusion in the toxicity evaluation. a-h Images of the heart (**a**), liver (**b**), spleen (**c**), lung (**d**), kidney (**e**), stomach (**f**), brain (**g**), and left femur (**h**), collected on day 8 post-CAR-T infusion for morphological analysis.

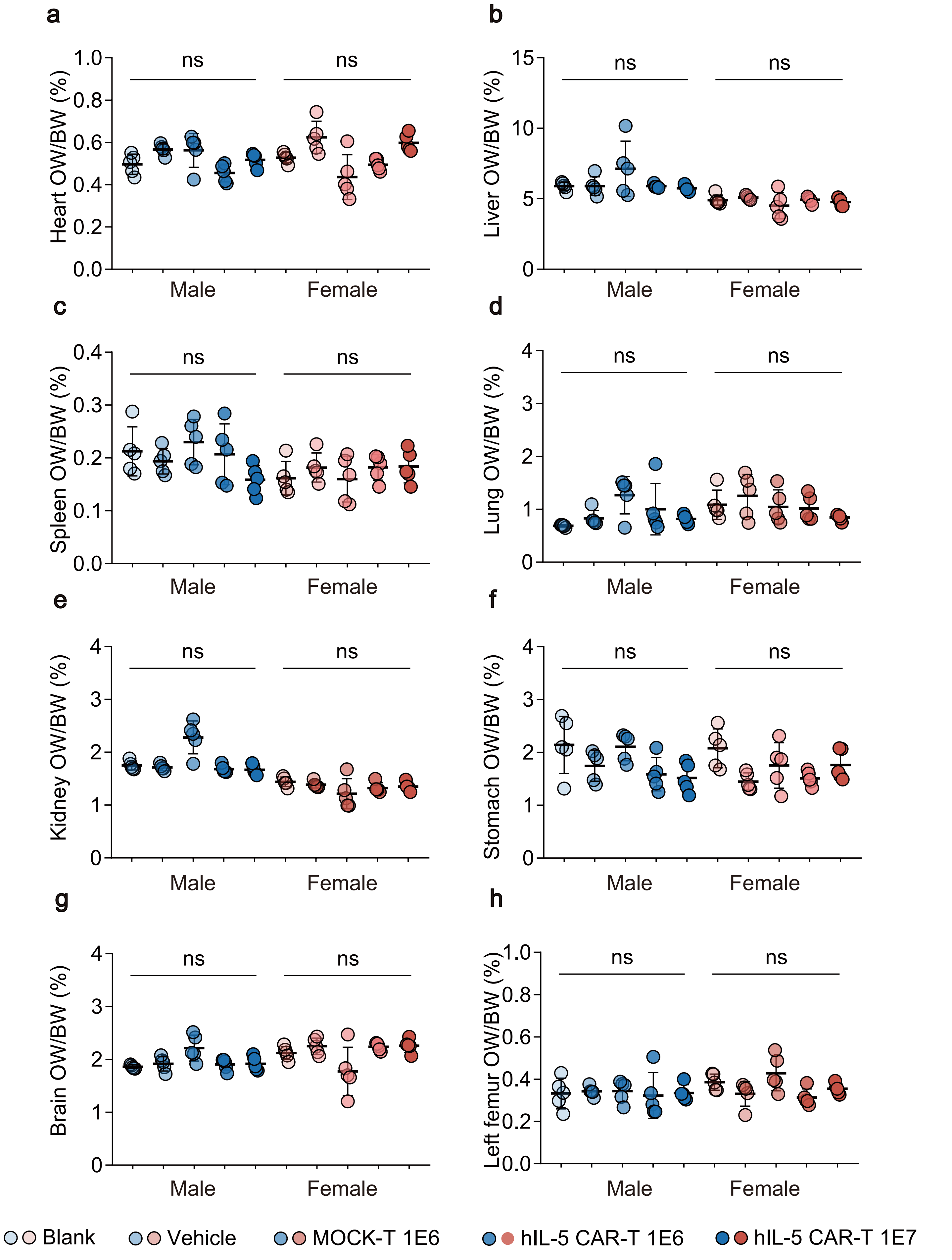

Suppl Fig6. Organ-to-body weight (OW/BW) ratios on day 8 post-CAR-T infusion in the toxicity evaluation. a-h Organ-to-body weight ratios of the heart (**a**), liver (**b**), spleen (**c**), lung (**d**), kidney (**e**), stomach (**f**), brain (**g**) and left femur (**h**), measured on day 8 post-CAR-T infusion. Data are presented as mean ± SD. *$P < 0.05$, **$P < 0.01$, ***$P < 0.001$, ****$P < 0.0001$; ns, not significant (two-way ANOVA).

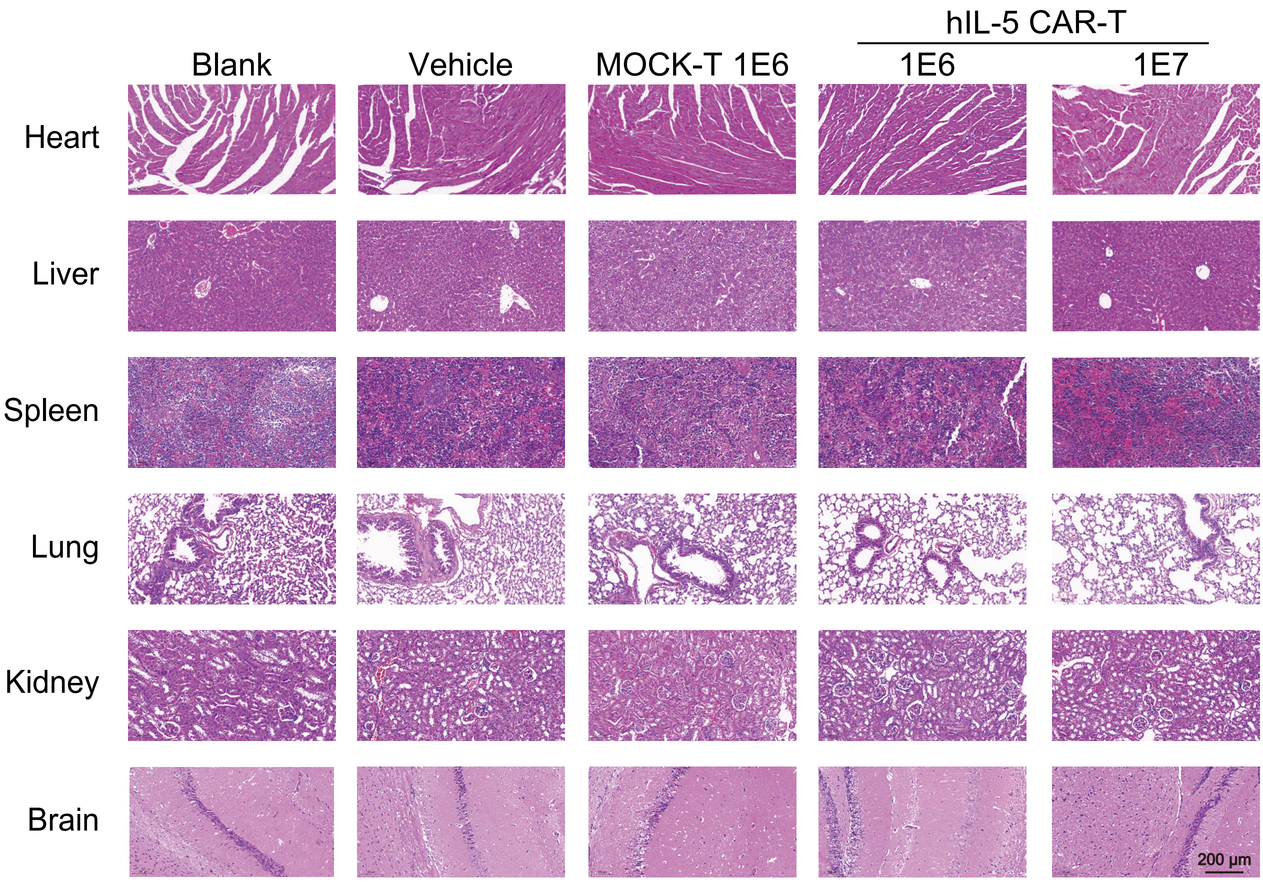

Suppl Fig7. Histological analysis of organs in the toxicity evaluation. H&E staining of organs (heart, liver, spleen, lung, kidney, and brain) collected on day 8 post-CAR-T infusion. Scale bars, 200 μm.

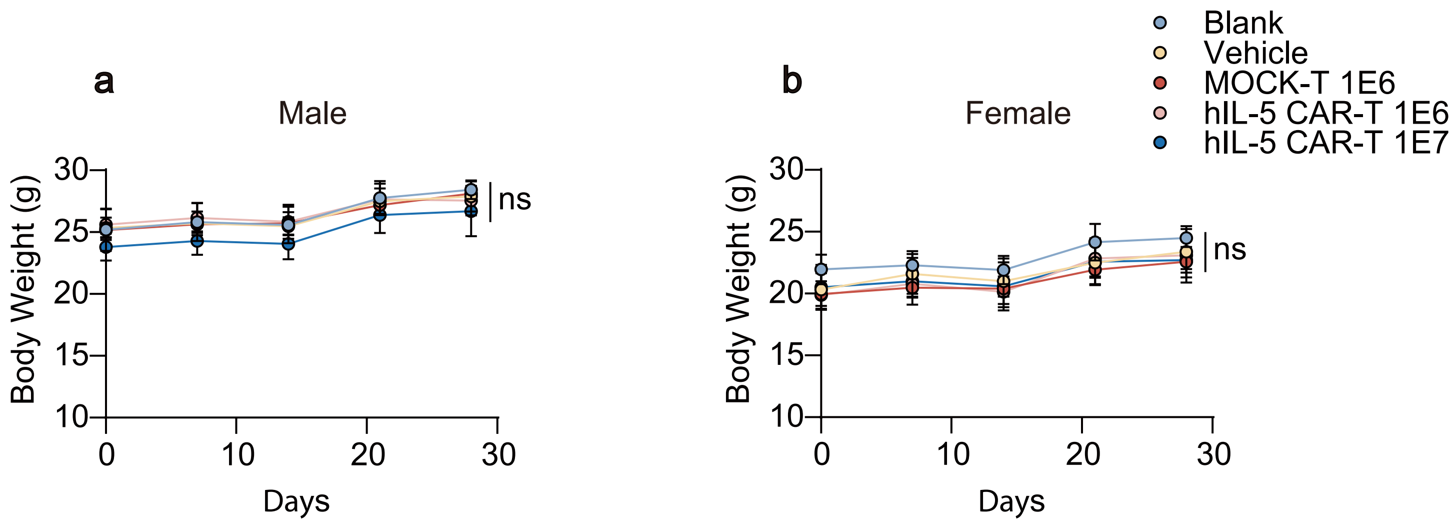

Suppl Fig8. Sex-dimorphic body weight responses in mice during 29-day toxicity study. a-b The weight change curves of male (**a**) and female (**b**) mice treated with blank, vehicle, MOCK-T (1×10^6), hIL-5 CAR-T (1×10^6), or hIL-5 CAR-T (1×10^7) over the course of the study. Data are presented as mean ± SD (n=10). *$P < 0.05$, **$P < 0.01$, ***$P < 0.001$, ****$P < 0.0001$; ns, not significant (two-way ANOVA).

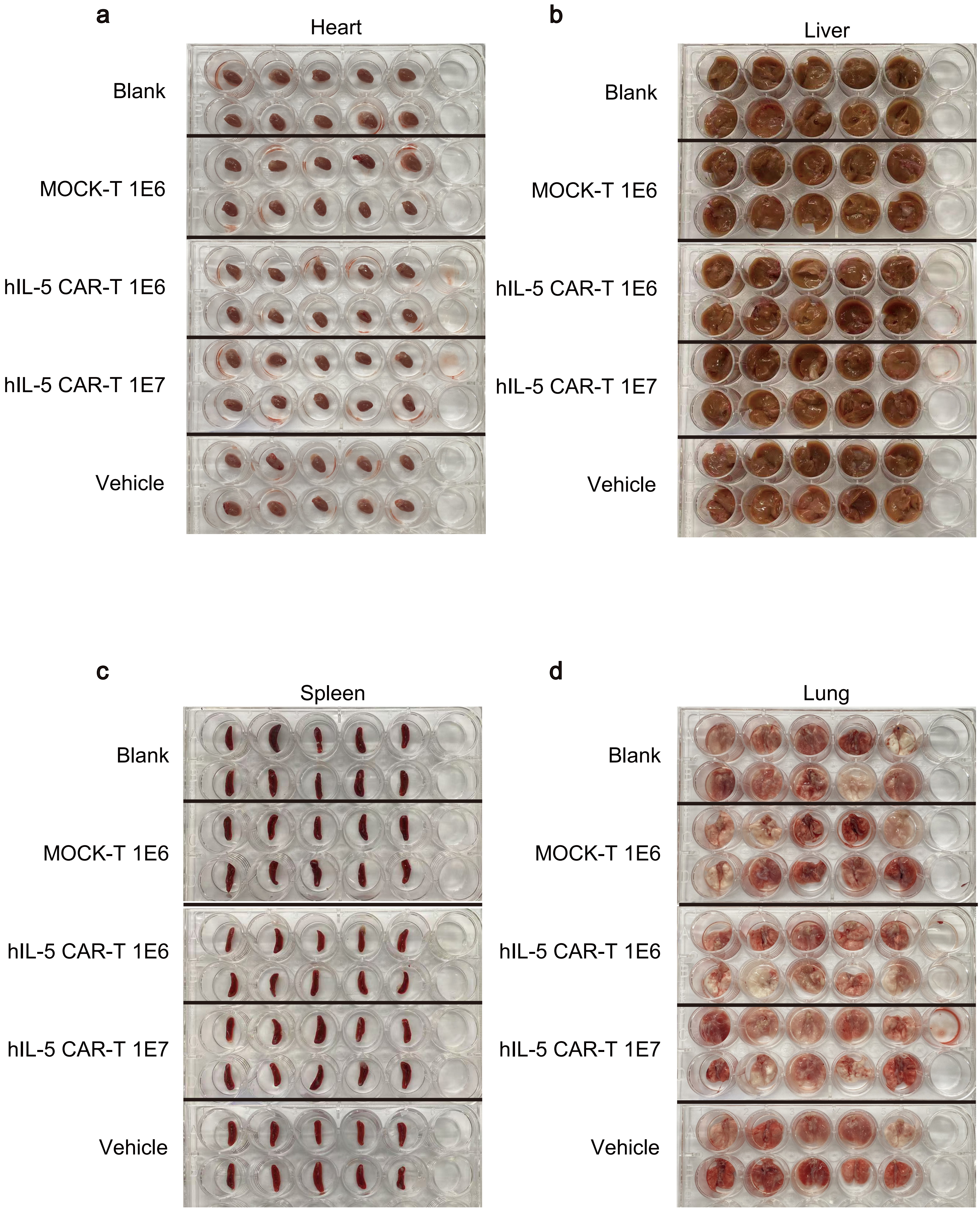
a
Heart
Blank
MOCK-T 1E6
hIL-5 CAR-T 1E6
hIL-5 CAR-T 1E7
Vehicle
b
Liver
Blank
MOCK-T 1E6
hIL-5 CAR-T 1E6
hIL-5 CAR-T 1E7
Vehicle
c
Spleen
Blank
MOCK-T 1E6
hIL-5 CAR-T 1E6
hIL-5 CAR-T 1E7
Vehicle
d
Lung
Blank
MOCK-T 1E6
hIL-5 CAR-T 1E6
hIL-5 CAR-T 1E7
Vehicle

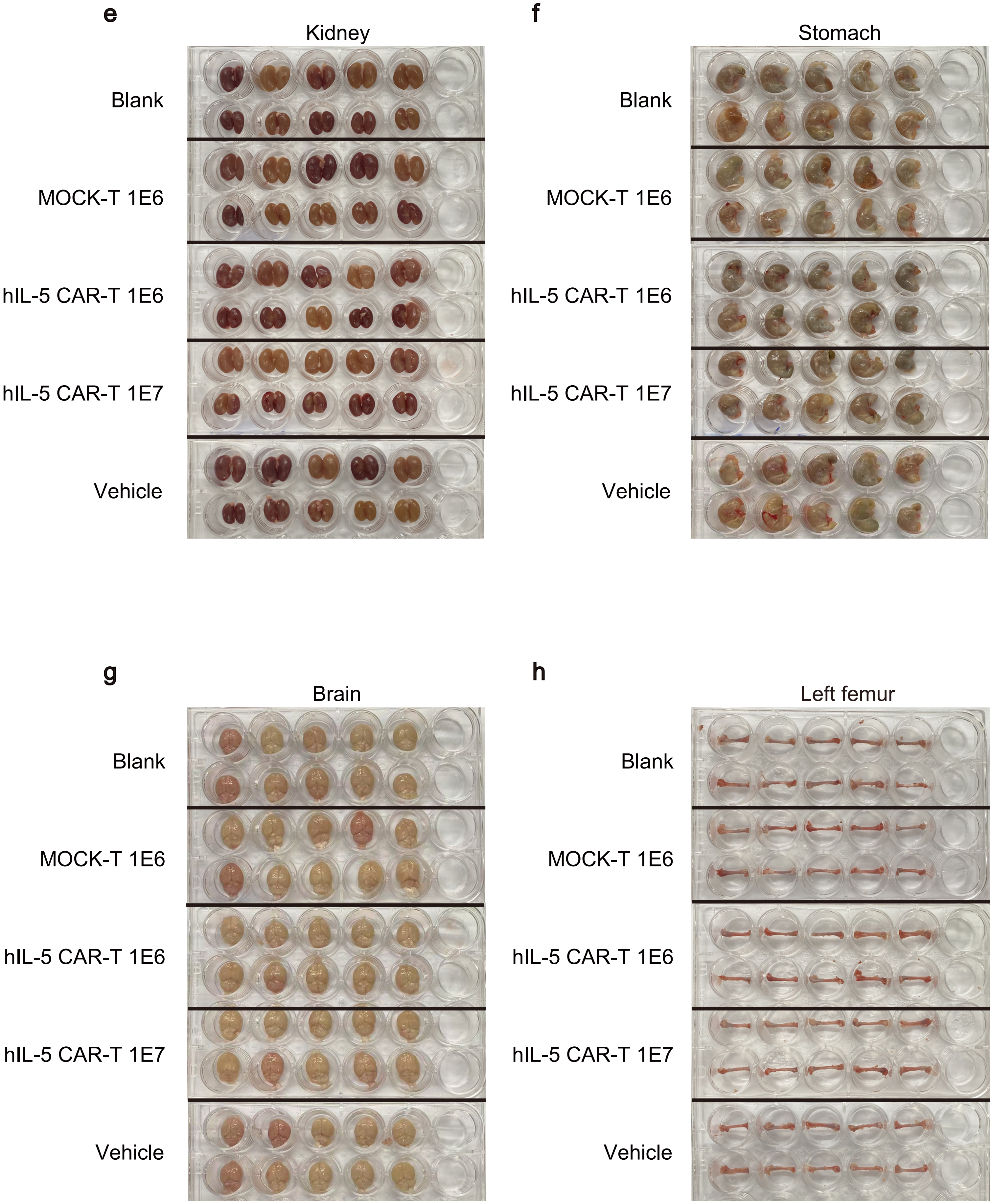

Suppl Fig9. Morphological analysis of collected organs on day 29 post-CAR-T infusion in the toxicity evaluation. a-h Images of the heart (**a**), liver (**b**), spleen (**c**), lung (**d**), kidney (**e**), stomach (**f**), brain (**g**), and left femur (**h**), collected on day 29 post-CAR-T infusion for morphological analysis.

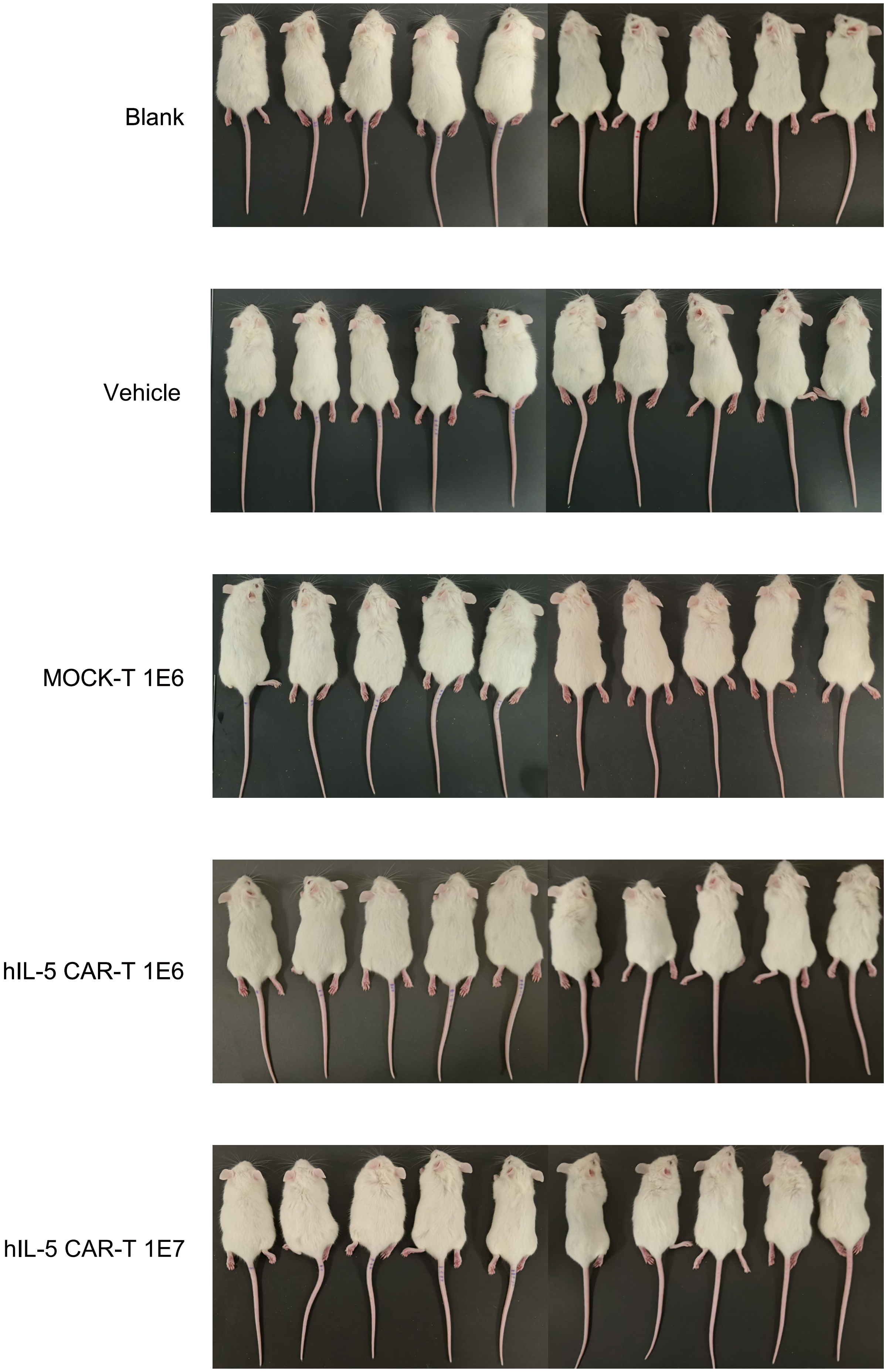

Suppl Fig10. Injection site evaluation on day 29 post-CAR-T infusion in the toxicity evaluation. Images of the tail injection site in NCG mice on day 29 post-CAR-T infusion are shown. No signs of infection, hematoma, or skin thickening were observed.

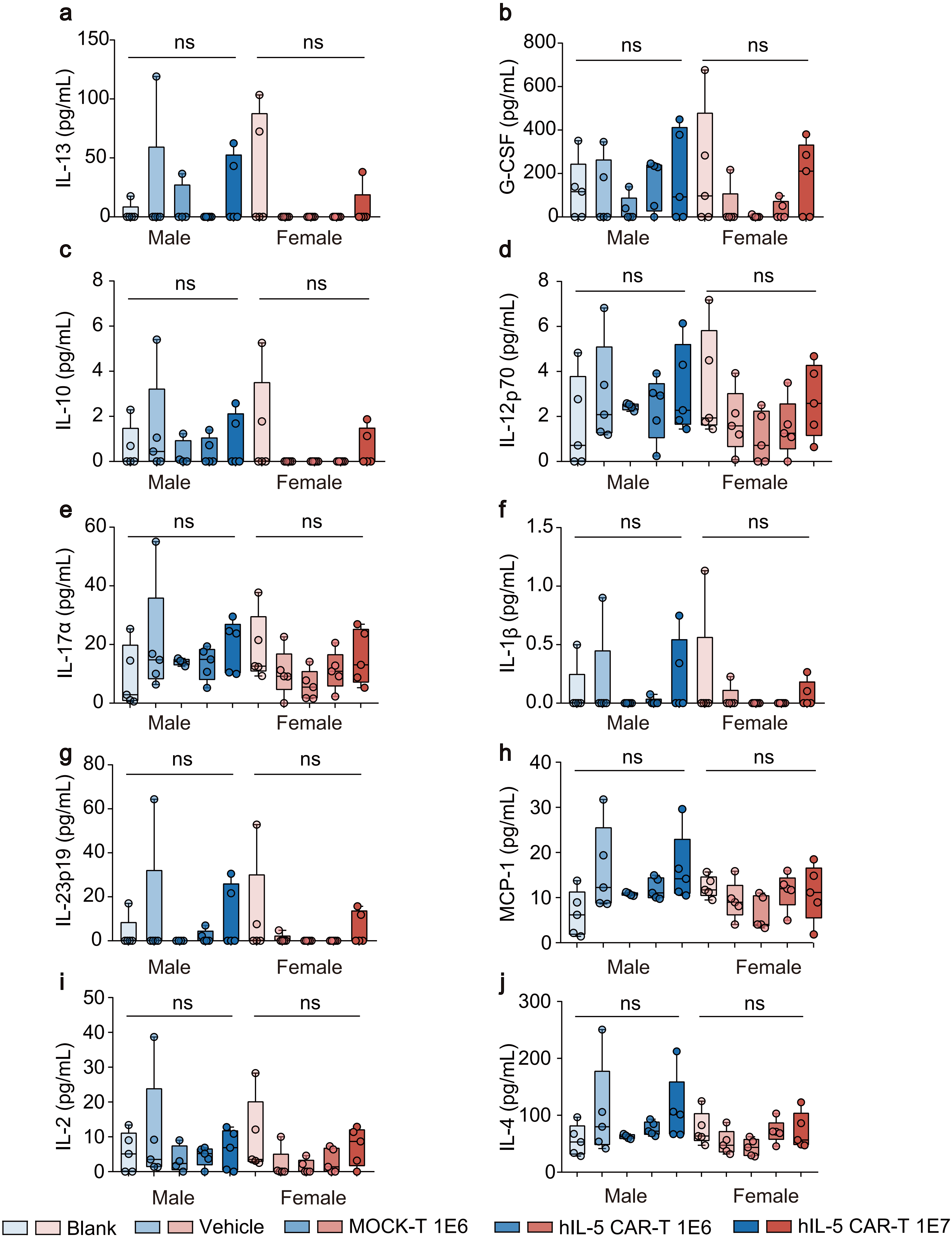

Suppl Fig11. Human serum cytokine levels on day 8 post-CAR-T infusion in the toxicity evaluation. a-j Human serum cytokine levels of IL-13 (**a**), G-CSF (**b**), IL-10 (**c**), IL-12p70 (**d**), IL-17α (**e**), IL-1β (**f**), IL-23p19 (**g**), MCP-1 (**h**), IL-2 (**i**) and IL-4 (**j**) measured on day 8 post-CAR-T infusion. **P* < 0.05, ***P* < 0.01, ****P* < 0.001, *****P* < 0.0001; ns, not significant (one-way ANOVA).

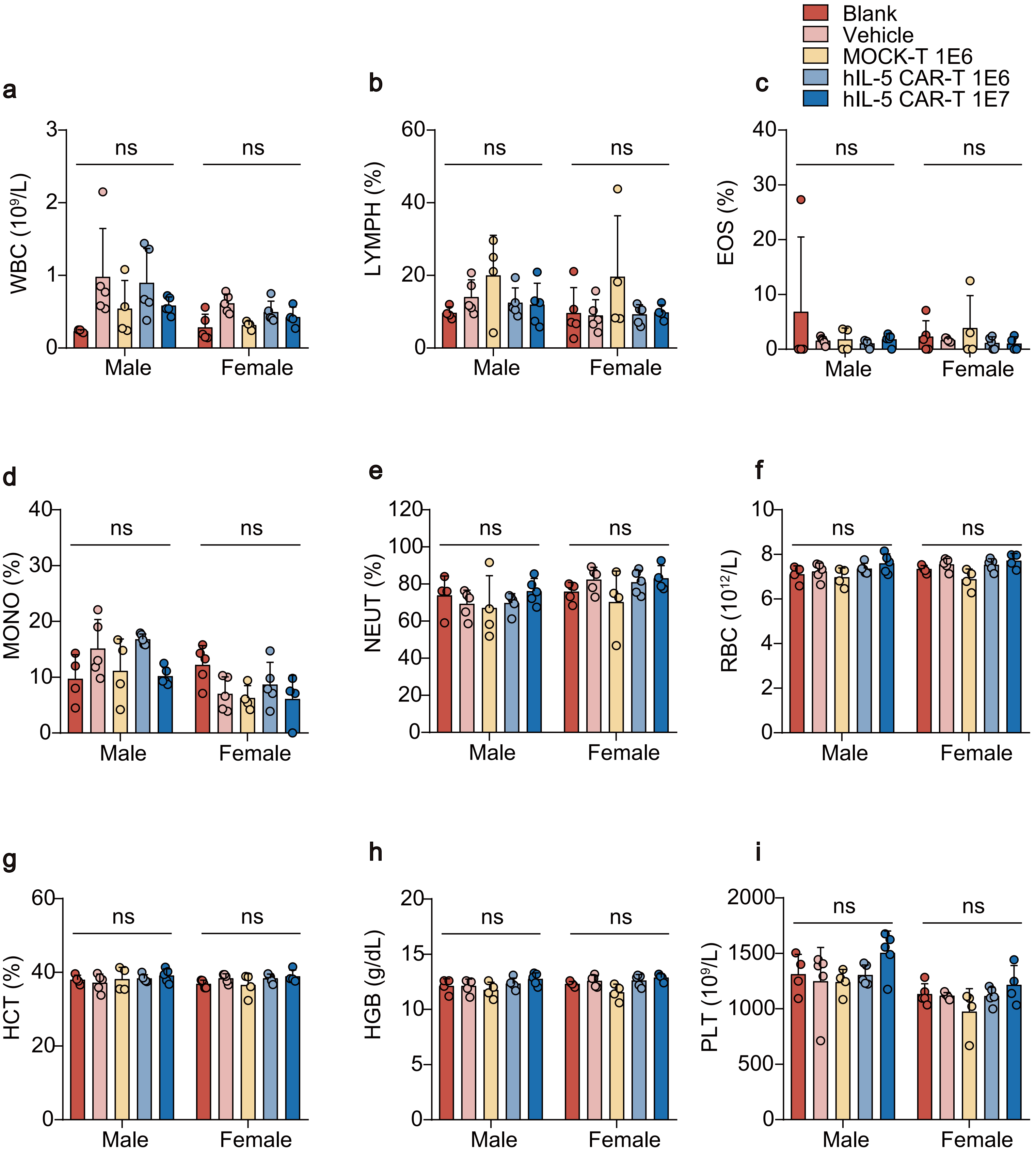

Suppl Fig12. Hematological analysis on day 8 post-CAR-T infusion in the toxicity evaluation. a–i Complete blood count (CBC) analysis conducted on day 8 post-CAR-T infusion to evaluate immune cell-associated hematologic toxicity (ICAHT) following CAR-T cell treatment, including white blood cell count (WBC, **a**), lymphocyte percentage (LYMPH, **b**), eosinophil percentage (EOS, **c**), monocyte percentage (MONO, **d**), neutrophil percentage (NEUT, **e**), red blood cell count (RBC, **f**), hematocrit (HCT, **g**), hemoglobin levels (HGB, **h**), and platelet count (PLT, **i**). *$P < 0.05$, **$P < 0.01$, ***$P < 0.001$, ****$P < 0.0001$; ns, not significant (two-way ANOVA).

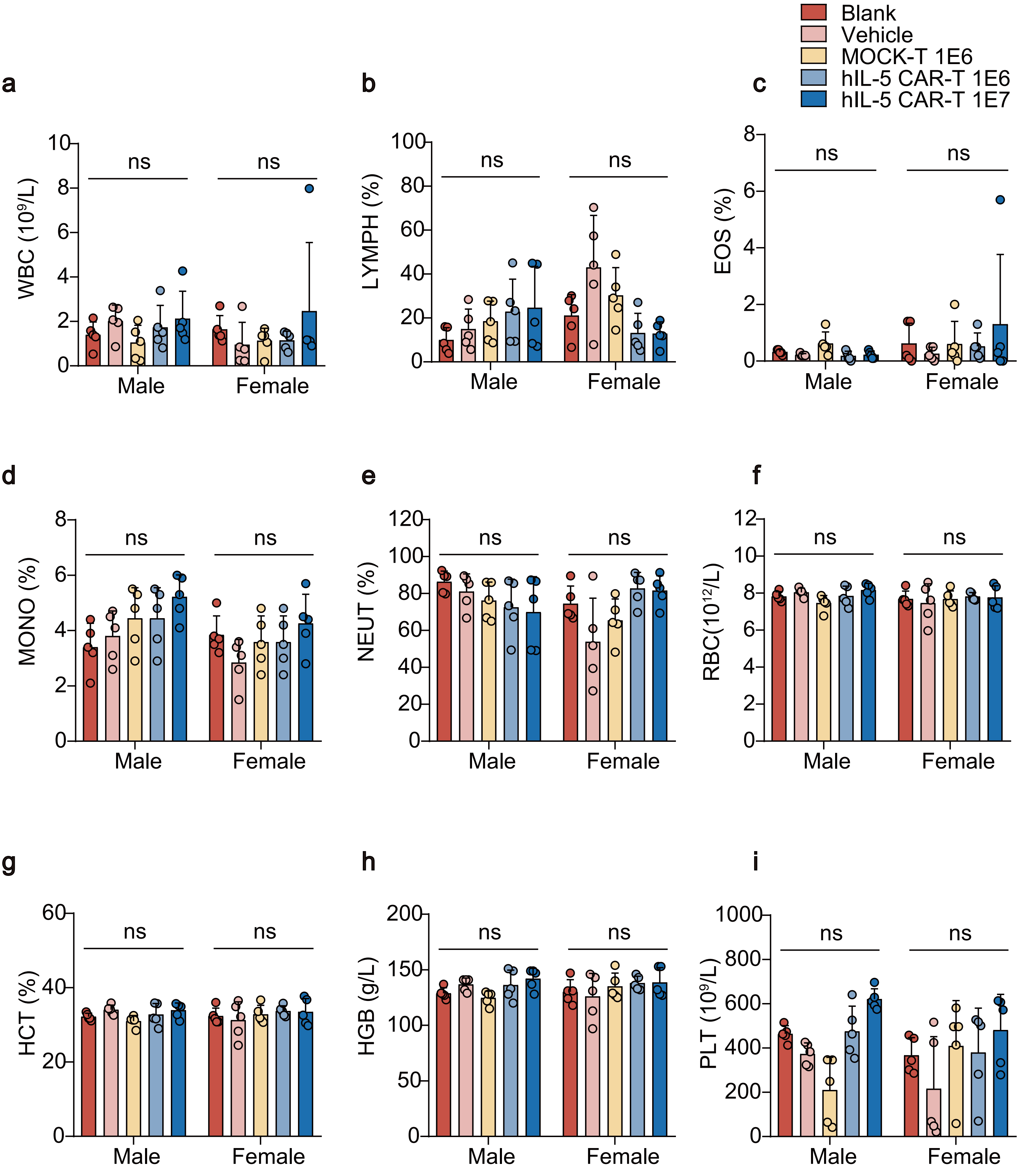

Suppl Fig13. Hematological analysis on day 29 post-CAR-T infusion in the toxicity evaluation. a–i CBC analysis conducted on day 29 post-CAR-T infusion to evaluate ICAHT following CAR-T cell treatment, including white blood cell count (WBC, **a**), lymphocyte percentage (LYMPH, **b**), eosinophil percentage (EOS, **c**), monocyte percentage (MONO, **d**), neutrophil percentage (NEUT, **e**), red blood cell count (RBC, **f**), hematocrit (HCT, **g**), hemoglobin levels (HGB, **h**), and platelet count (PLT, **i**). $*P < 0.05$, $**P < 0.01$, $***P < 0.001$, $****P < 0.0001$; ns, not significant (two-way ANOVA).

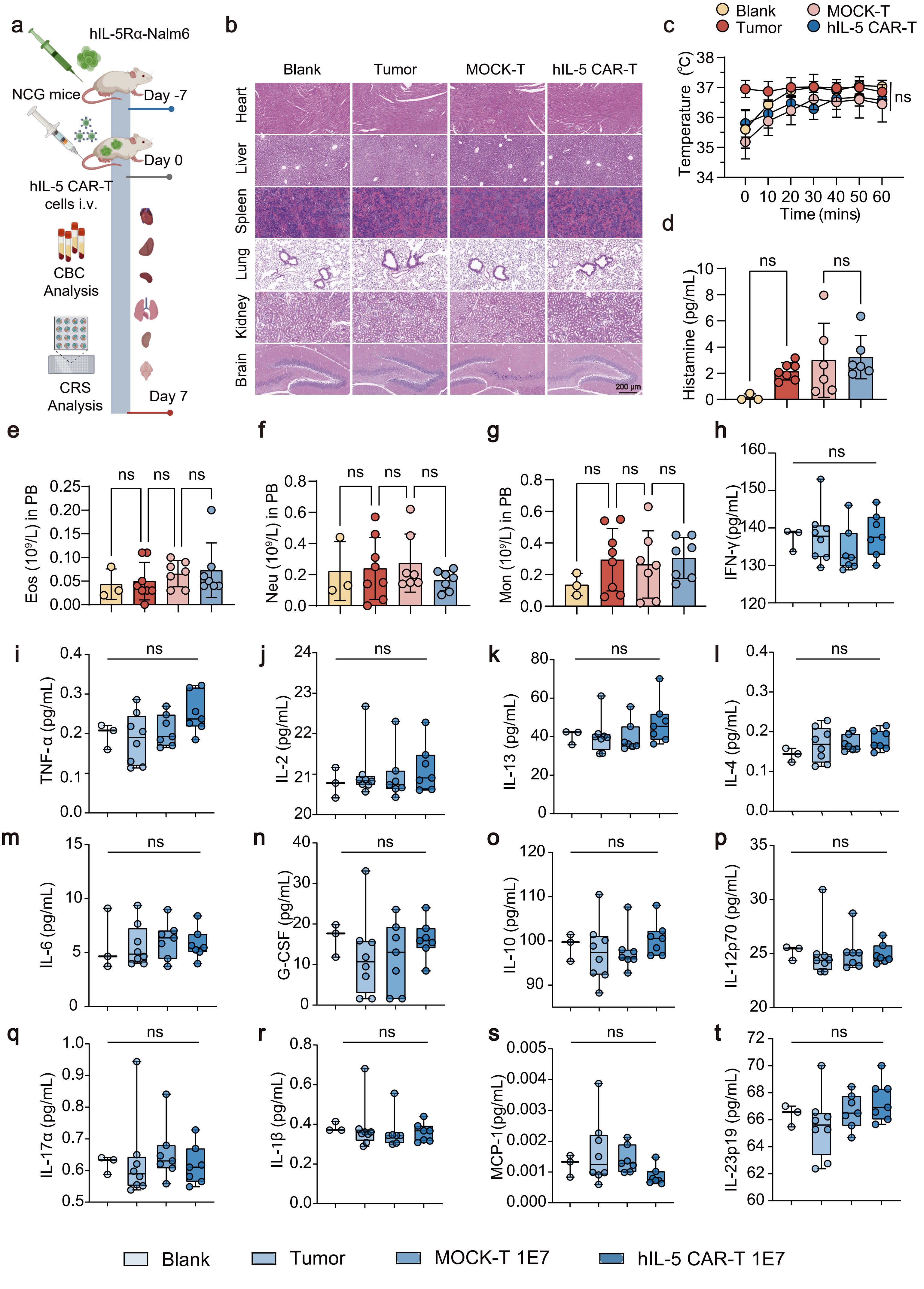
a
hIL-5Rα-Nalm6
NCG mice
Day -7
Day 0
hIL-5 CAR-T cells i.v.
CBC Analysis
CRS Analysis
Day 7
b
Blank
Tumor
MOCK-T
hIL-5 CAR-T
Heart
Liver
Spleen
Lung
Kidney
Brain
200 μm
c
Blank
Tumor
MOCK-T
hIL-5 CAR-T
Temperature (℃)
Time (mins)
ns
d
Histamine (pg/mL)
ns
e
Eos (10⁹/L) in PB
f
Neu (10⁹/L) in PB
g
Mon (10⁹/L) in PB
h
IFN-γ (pg/mL)
i
TNF-α (pg/mL)
j
IL-2 (pg/mL)
k
IL-13 (pg/mL)
l
IL-4 (pg/mL)
m
IL-6 (pg/mL)
n
G-CSF (pg/mL)
o
IL-10 (pg/mL)
p
IL-12p70 (pg/mL)
q
IL-17α (pg/mL)
r
IL-1β (pg/mL)
s
MCP-1 (pg/mL)
t
IL-23p19 (pg/mL)
Blank
Tumor
MOCK-T 1E7
hIL-5 CAR-T 1E7

Suppl Fig14. In vivo safety assessment of hIL-5 CAR-T cells in a hypereosinophilic leukemia model. a Schematic of the experimental design: hIL-5Rα-Nalm6 engraftment (Day -7) followed by hIL-5 CAR-T (1×10^7) and MOCK-T infusion (Day 0), with analyses (CBC, CRS mediators) at Day 7. **b** H&E staining of major organs (scale bar: 200 μm) showing preserved histoarchitecture across groups. **c** Body temperature was monitored after intravenous injection of hIL-5 CAR-T or MOCK-T cells. **d–t** Peripheral blood levels of allergy-associated immune cells and cytokines in CRS. No significant differences were observed in peripheral blood immune cell counts, including eosinophils (**e**), neutrophils (**f**), monocytes (**g**), between groups. Similarly, circulating levels of allergy-associated mediators and cytokines, including histamine (**d**), IFN-γ (**h**), TNF-α (**i**), IL-2 (**j**), IL-13 (**k**), IL-4 (**l**), IL-6 (**m**), G-CSF (**n**), IL-10 (**o**), IL-12p70 (**p**), IL-17α (**q**), IL-1β (**r**), MCP-1 (**s**), and IL-23p19 (**t**), were comparable between groups, with no statistically significant differences. Data in panels (**c–t**) are shown as mean ± SD. P values were calculated by two-way ANOVA (**c-g**) or one-way ANOVA (**h-t**); $^*P < 0.05$, $^{**}P < 0.01$, $^{***}P < 0.001$, $^{****}P < 0.0001$; ns, not significant.

a

cluster	cell types	cluster	cell types	cluster	cell types	cluster	cell types
C01	Basophils	C09	DNT	C17	Neutrophils	C25	Neutrophils
C02	NK cells	C10	CD8T	C18	Neutrophils	C26	CD4T
C03	NK cells	C11	DNT	C19	Neutrophils	C27	CD8T
C04	CD4T	C12	CD8T	C20	Neutrophils	C28	B cells
C05	CD4T	C13	CD8T	C21	Neutrophils	C29	Monocytes
C06	CD4T	**C14**	**Eosinophils**	C22	Neutrophils	C30	Monocytes
C07	CD4T	C15	B cells	C23	Neutrophils	C31	Monocytes
C08	DNT	C16	Others	C24	Neutrophils	C32	Monocytes

b

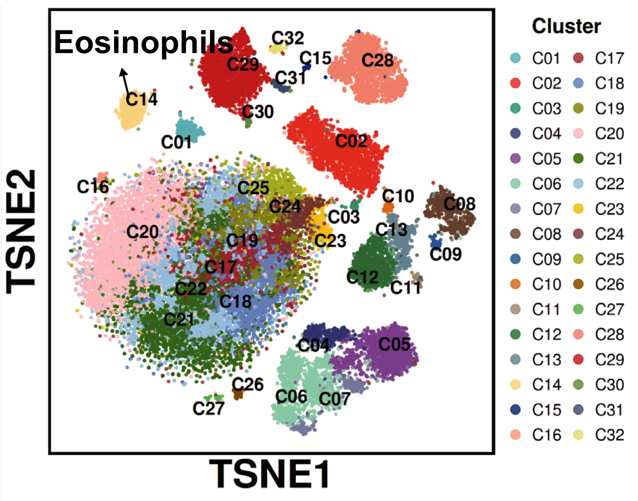

c

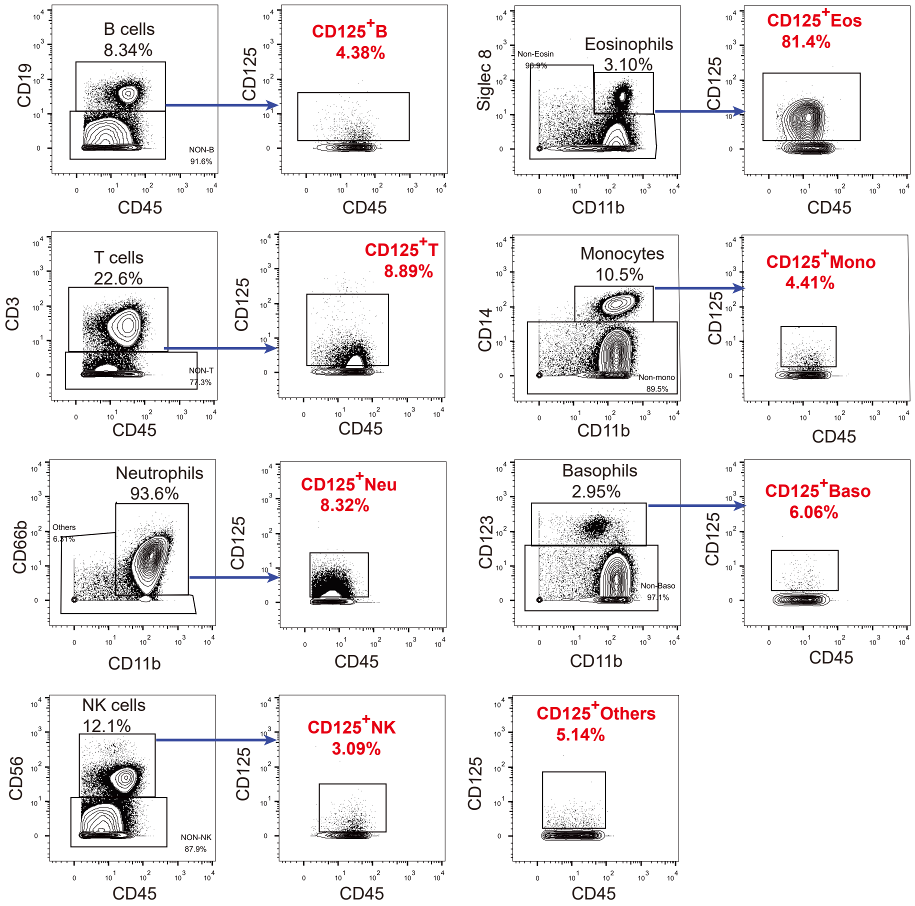

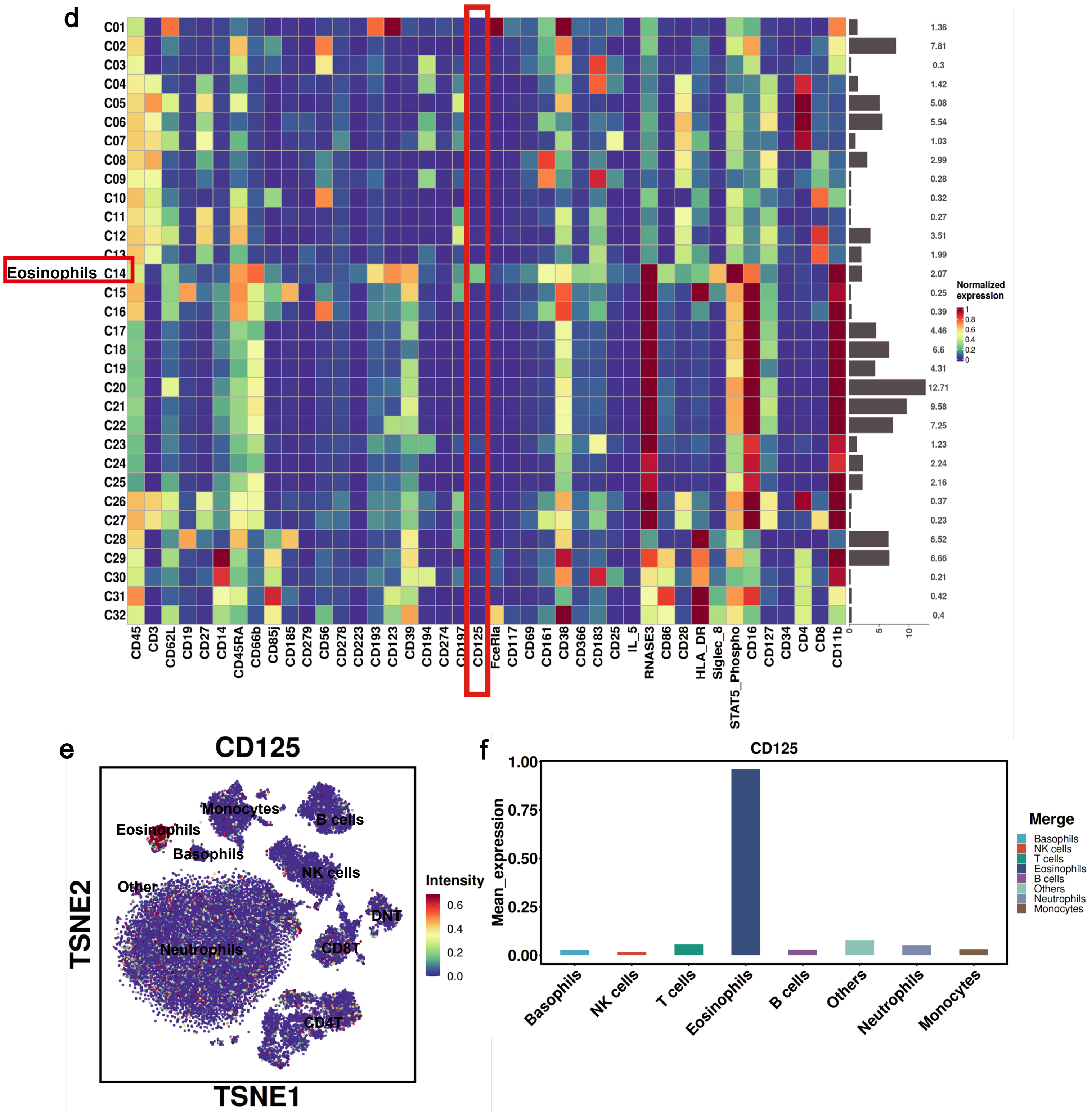

Suppl Fig15. Single-cell cluster annotation and CD125 (IL-5Rα) expression profile in peripheral blood. a Summary table showing the annotation of clusters (C01–C32) derived from peripheral blood into major cell types, with cluster C14 identified as eosinophils. **b** t-SNE projection of all cells colored by cluster identity, with major cell populations labeled; the eosinophil cluster is indicated by an arrow. **c** Representative flow cytometry gating of major immune populations from peripheral blood with quantification of CD125 positive cells, confirming predominant CD125 expression in eosinophils. **d** Heatmap of normalized marker expression across clusters C01–C32. Cluster C14 (eosinophils) and CD125 are highlighted, showing selective enrichment in eosinophils. **e** Feature plot showing CD125 expression overlaid on the t-SNE map of peripheral blood cells, where color intensity (blue to red) represents low to high expression levels, revealing enriched CD125 expression in eosinophils. **f** Bar plot of mean CD125 expression across annotated cell types.

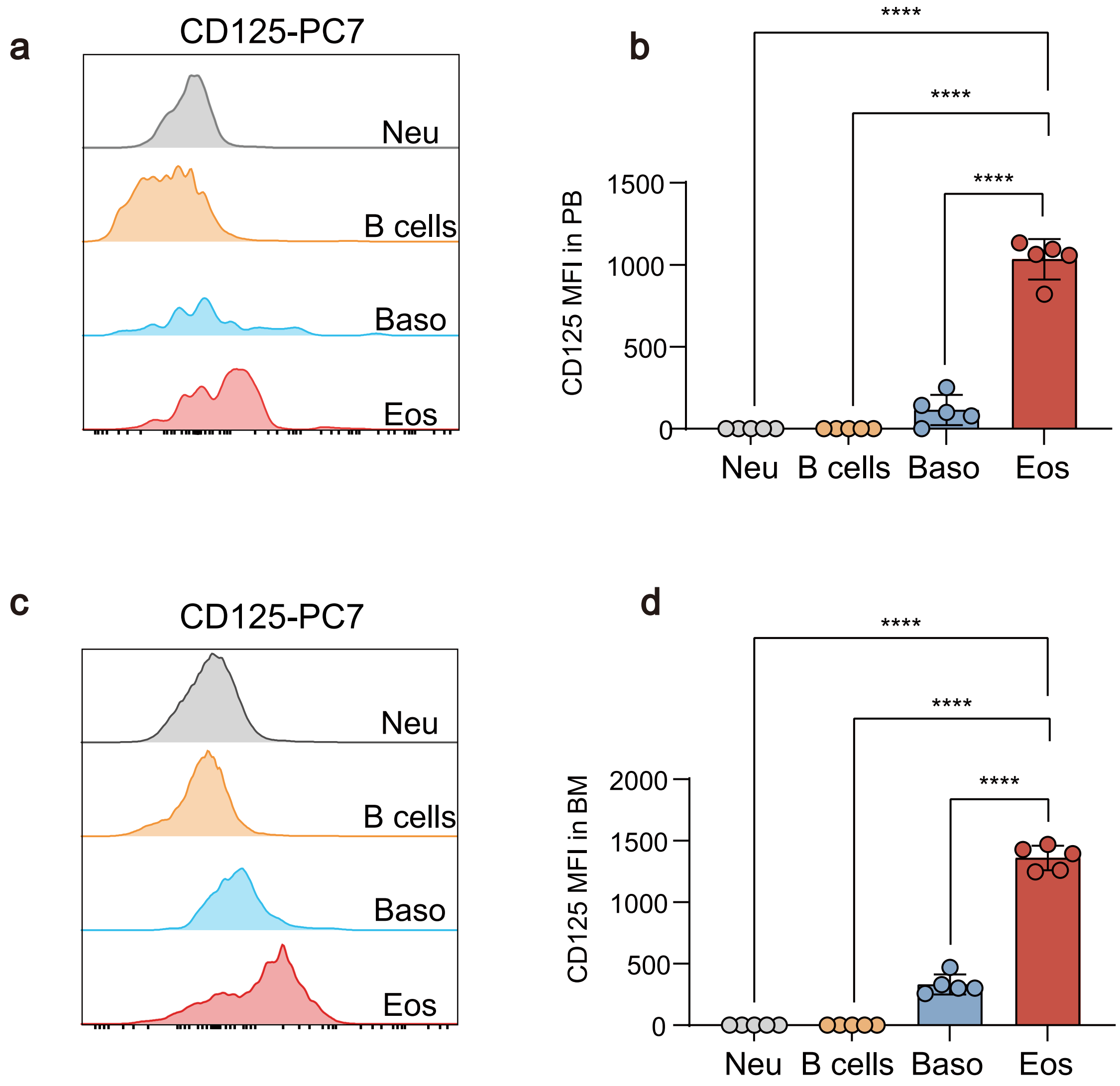

Suppl Fig16. Expression of CD125 on immune cell subsets in mouse peripheral blood (PB) and bone marrow (BM). a,c Flow cytometric histograms showing CD125 expression on neutrophils (Neu), B cells, basophils (Baso) and eosinophils (Eos) in PB (**a**) and BM (**c**). **b,d** Quantification of CD125 median fluorescence intensity (MFI) in PB (**b**) and BM (**d**) for the indicated immune cell populations (n=5). Data are shown as mean ± SD, *$P < 0.05$, **$P < 0.01$, ***$P < 0.001$, ****$P < 0.0001$; ns, not significant (two-way ANOVA).

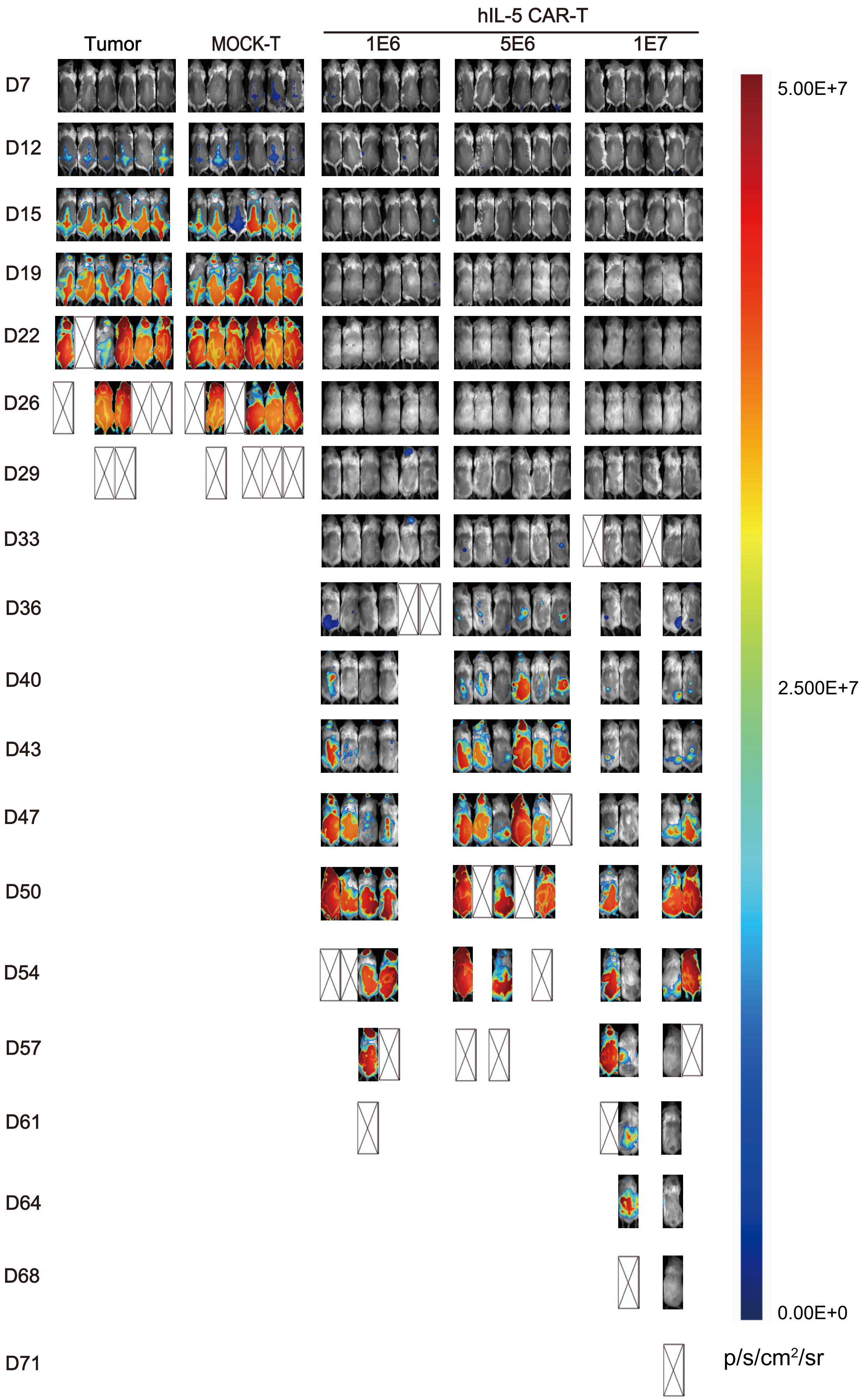

Suppl Fig17. Anti-tumor efficacy of hIL-5 CAR-T cells in a hypereosinophilic leukemia model. Schematic of the in vivo antitumor efficacy study (full version corresponding to Figure. **6b**). NCG mice were intravenously injected with hIL-5Rα–Nalm6 cells and treated on day 7 with MOCK-T (1×10^6) or escalating doses of hIL-5 CAR-T cells (1×10^6, 5×10^6, or 1×10^7; *n*=6). Tumor burden was assessed by bioluminescence imaging. "×" indicates mice reaching the ethical endpoint.

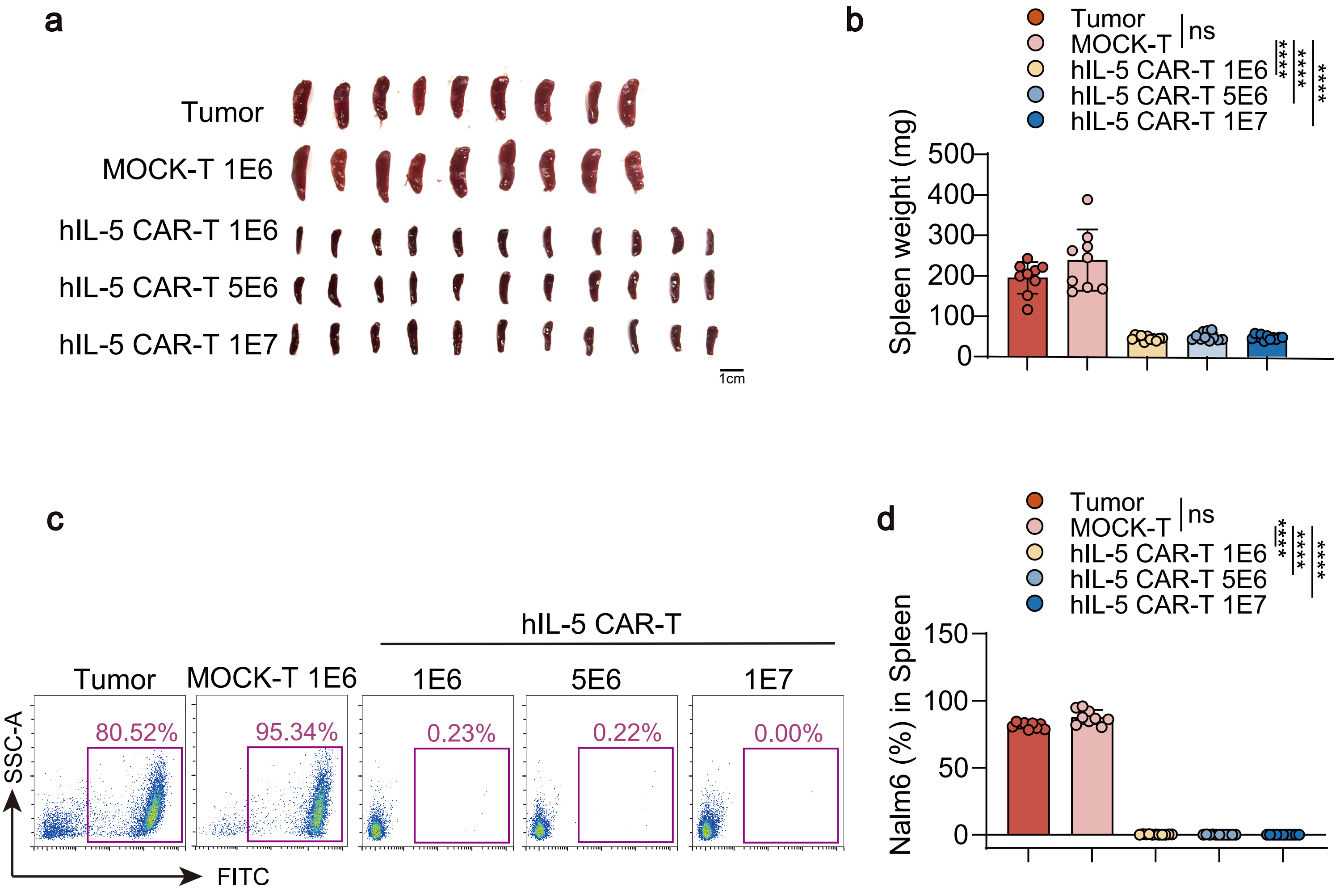

Suppl Fig18. Therapeutic efficacy assessment in a hypereosinophilic leukemia model. a Representative images of spleens harvested from tumor-bearing mice treated with MOCK-T cells or escalating doses of hIL-5 CAR-T cells (1×10^6, 5×10^6, or 1×10^7). Scale bar, 1 cm. **b** Quantification of spleen weight in the indicated treatment groups. **c** Representative flow cytometry plots showing the frequency of EGFP-transduced Nalm6 tumor cells in the spleen, identified by FITC-channel fluorescence. **d** Quantification of Nalm6 tumor burden in the spleen across groups (n=9–11). Data are presented as mean ± SD. *$P < 0.05$, **$P < 0.01$, ***$P < 0.001$, ****$P < 0.0001$; ns, not significant (two-way ANOVA).

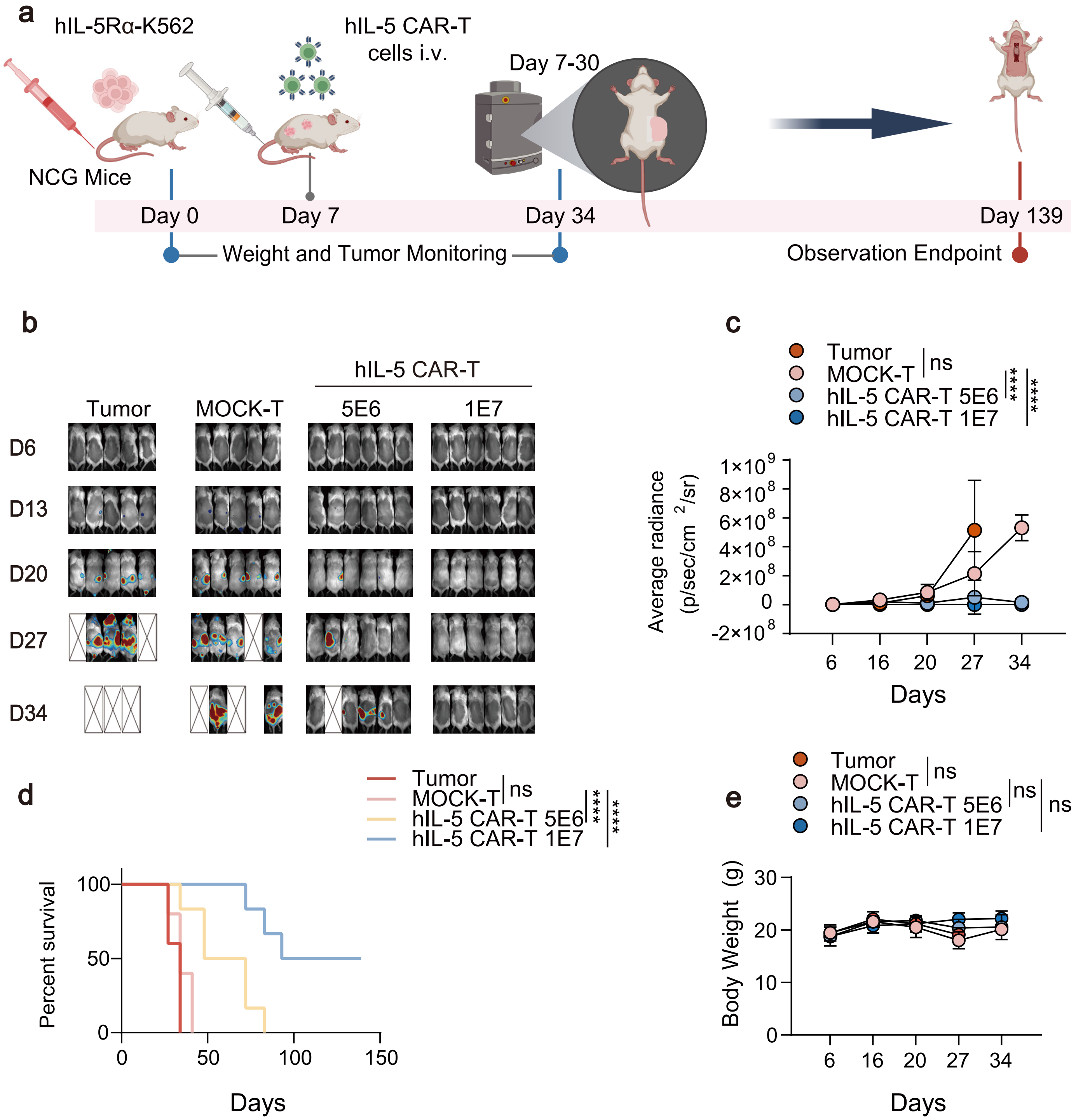

Suppl Fig19. Anti-tumor efficacy of hIL-5 CAR-T cells in a hypereosinophilic leukemia model. a Schematic representation of the in vivo study assessing the anti-tumor activity of hIL-5 CAR-T cells. NCG mice were intravenously injected with hIL-5Rα-K562 cells and randomly assigned to four groups. On day 7, mice received MOCK-T cells or hIL-5 CAR-T cells at doses of 5×10^6 or 1×10^7 cells, as indicated (*n*=5–6). Tumor burden was monitored by bioluminescence imaging, along with body weight and survival curve assessments. Elements were created using BioRender.com. **b** Bioluminescence imaging of tumor burden at the indicated time points following CAR-T cell infusion. "×" indicates mice euthanized upon reaching the ethical endpoint. **c** Quantification of leukemia burden based on average bioluminescence intensity from (**b**) (*n*=5–6). **d** Kaplan-Meier survival curves depicting overall survival. **e** Body weight curves of mice from (**a**). $^*P < 0.05$, $^{**}P < 0.01$, $^{***}P < 0.001$, $^{****}P < 0.0001$; ns, not significant (two-way ANOVA; **c, e**).

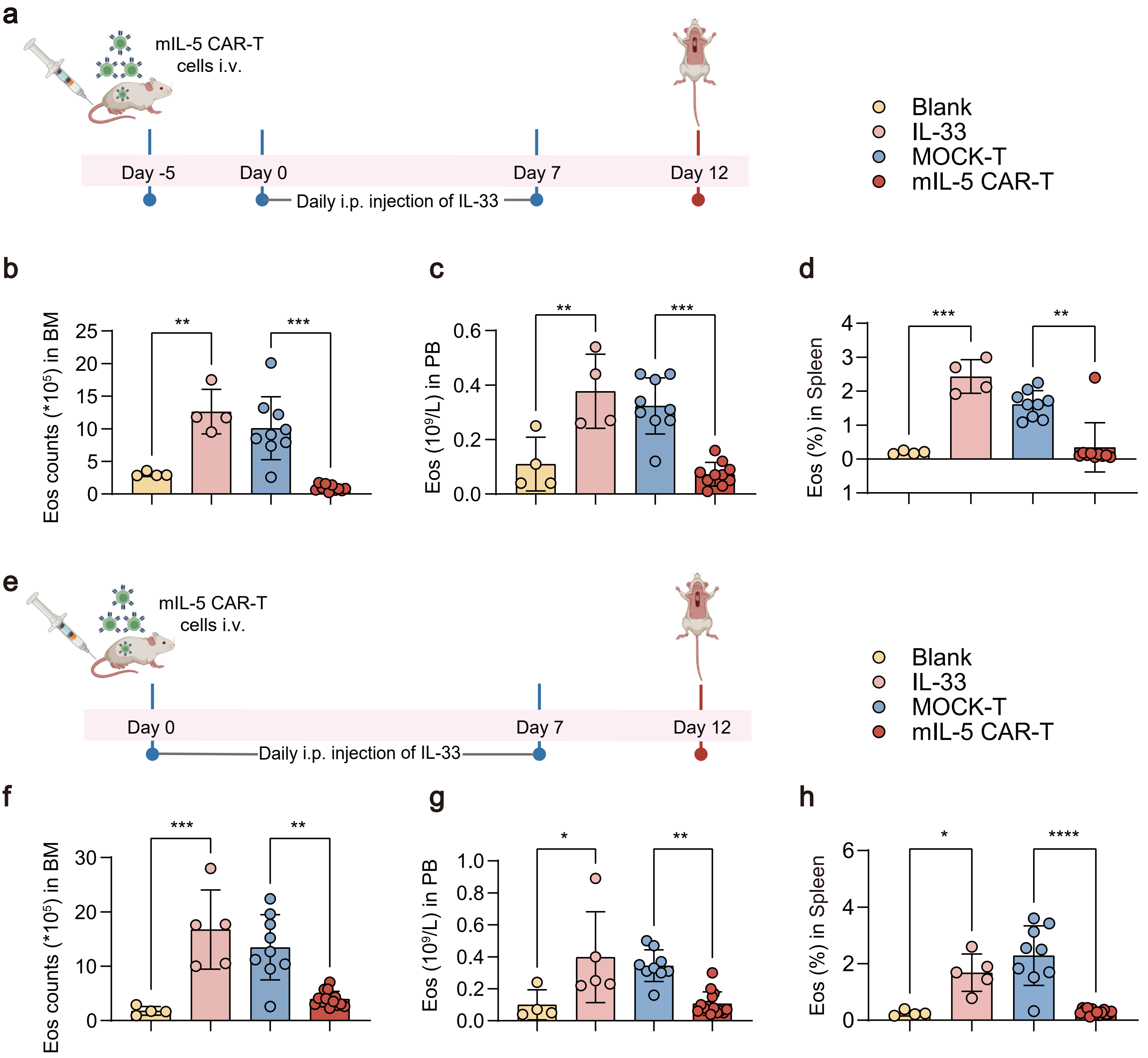

Suppl Fig20. Therapeutic efficacy of mIL-5 CAR-T cells in the IL-33-induced eosinophilia model. a Schematic of the in vivo experimental design. Primary T cells from BALB/c mice were transduced with mIL-5 CAR comprising a mouse IL-5 linked to mouse CD28 costimulatory and CD3ζ signaling domains. Mice received intravenous infusion of mIL-5 CAR-T cells (3×10^6) or MOCK-T cells on day −5 (prophylactic setting), followed by daily intraperitoneal administration of IL-33 starting on day 0. Tissues were collected on day 12. **b–d** Quantification of eosinophils in BM (**b**), PB (**c**), and spleen (**d**) from control mice and IL-33-induced mice receiving MOCK-T or mIL-5 CAR-T cells. **e** Schematic of the in vivo experimental design. Mice received intravenous infusion of mIL-5 CAR-T cells (3×10^6) or MOCK-T cells on day 0 (therapeutic setting), followed by daily intraperitoneal administration of IL-33 starting on day 0. Tissues were collected on day 12. **f–h** Quantification of eosinophils in BM (**f**), PB (**g**), and spleen (**h**) from control mice and IL-33-induced mice receiving MOCK-T or mIL-5 CAR-T cells. Elements were created using BioRender.com. Data are presented as mean ± SD (n = 4–14). *$P < 0.05$, **$P < 0.01$, ***$P < 0.001$, ****$P < 0.0001$; ns, not significant (two-way ANOVA).
